# The prognostic value of additional copies of 1q21 in multiple myeloma depends on the primary genetic event

**DOI:** 10.1002/ajh.25994

**Published:** 2020-09-19

**Authors:** Maurus Locher, Michael Steurer, Emina Jukic, Markus A. Keller, Friedrich Fresser, Carmen Ruepp, Ewald Wöll, Irmgard Verdorfer, Günther Gastl, Wolfgang Willenbacher, Roman Weger, David Nachbaur, Dominik Wolf, Eberhard Gunsilius, Johannes Zschocke, Normann Steiner

**Affiliations:** ^1^ Institute of Human Genetics Medical University of Innsbruck Innsbruck Austria; ^2^ Department of Internal Medicine V Medical University of Innsbruck Innsbruck Austria; ^3^ Department of Internal Medicine St. Vinzenz Krankenhaus Betriebs GmbH Zams Austria; ^4^ ONCOTYROL ‐ Center for Personalized Cancer Medicine Innsbruck Austria; ^5^ Medical Clinic 3, Oncology, Hematology, Immuno‐Oncology and Rheumatology University Hospital Bonn Bonn Germany

## Abstract

Hyperdiploidy (HRD) and specific immunoglobulin heavy locus (*IGH*) translocations are primary chromosomal abnormalities (CA) in multiple myeloma (MM). In this retrospective study of 794 MM patients we aimed to investigate clinical features and common CA including gain(1q) in separate subgroups defined by primary CA. In the entire group, we confirmed that gain(1q) was associated with short time to next treatment and adverse overall survival (OS). The impact was worse for four or more copies of 1q21 as compared to three copies. However, in a subgroup of patients with clonal gain(11q) and without known primary *IGH* translocations (CG11q), already three copies of 1q21 were associated with a poor outcome; in the absence of gain(1q), patients in this subgroup had a remarkably long median OS of more than nine years. These cases were associated with HRD, coexpression of CD56 and CD117, male gender, and IgG subtype. In non‐CG11q patients, four or more copies of 1q21 (but not three copies) had a significant adverse impact on outcome. Several associations with CA and clinical findings were observed for the defined subgroups. As an example, we found a predominance of early tetraploidy, plasma cell leukemia, and female gender in the t(14;16) subgroup. Our results underscore the importance of subgrouping in MM.

## 1 INTRODUCTION

Although novel drugs have improved the management of multiple myeloma (MM), the disease is still characterized by a marked clinical heterogeneity as reflected by overall survival (OS), ranging from less than two years to more than ten years.^1^ Various factors such as patient fitness, therapy, microenvironment, and properties of the cancer itself including chromosomal abnormalities (CA) explain, at least in part, this heterogeneity.^2^, ^3^, ^4^, ^5^ With CA, MM can be broadly divided into two groups: about half of the cases with primary immunoglobulin heavy locus (IGH) translocations and the remaining with hyperdiploidy (HRD), the gain of odd‐number chromosomes.^6^ Both *IGH* translocations and HRD are considered primary genetic events, and as such they are mutually exclusive and present already in asymptomatic precursor stages and in the main clone.^7^ These initiating events are followed by secondary events that eventually contribute to tumor progression and relapse.^8^ In recent years, high‐throughput technologies such as gene expression profiling (GEP) and next‐generation sequencing (NGS) have been used to characterize myelomas in more detail in order to improve our understanding of myelomagenesis.^9^ Although another layer of complexity (eg, by showing clonal heterogeneity or many genes with recurrent mutations at low prevalence) was added, particularly by NGS, these studies also confirmed the importance of primary CA (ie, HRD and primary translocations) that define cytogenetic subgroups and give rise to a non‐random accumulation of secondary events.^10^, ^11^, ^12^ Fluorescence in situ hybridization (FISH) is implemented in standard clinical workflows for the detection of CA in order to identify high‐risk patients.^13^, ^14^, ^15^, ^16^ Several CA, namely primary *IGH* translocations and secondary events, have been associated with adverse prognosis. However, binary risk stratification based on the presence or absence of high‐risk CA might be oversimplified, and a possible explanation for heterogeneous survival of high‐risk patients.^4^ Recently, several new high‐risk groups were defined based on additional markers, co‐occurrence of adverse CA and weighted CA.^4^, ^17^, ^18^, ^19^, ^20^ Moreover, also the copy number (CN) of chromosomal gains might be associated with prognosis; the negative impact of gain(1q) on survival seems to be driven by the number of additional copies.^18^, ^21^, ^22^, ^23^ To define the impact of CN of common CA on clinical outcome in MM we here provide a detailed analysis of CA in the context of defined subgroups in a series of patients in the Austrian Myeloma Registry that were mainly treated with novel drugs and analyzed by FISH.

## 2 METHODS

### 2.1 Patients

Between January 2010 and February 2020, 1023 bone marrow (BM) and 4 peripheral blood samples from 794 patients who had a confirmed myeloma diagnosis with a plasma cell infiltration of ≥10% and/or one or more myeloma‐defining events^19^ were obtained to perform routine FISH analyses. Detailed clinical data including survival data were available for a subset of patients from the Austrian Myeloma Registry. The study was conducted in accordance with the Helsinki Declaration and approved by the local ethics committee of the Medical University of Innsbruck.

### 2.2 Interphase fluorescence in situ hybridization (FISH)

Interphase FISH analysis was performed on plasma cell‐enriched samples (344 patients) or unsorted samples (450 patients). Enrichment of CD138+ plasma cells was performed by either magnetic‐activated cell sorting (Miltenyi Biotec, Bergisch Gladbach, Germany) or with RoboSep (STEMCELL Technologies, Vancouver, Canada). Locus‐specific probes for the chromosomal regions 1q21 (*CKS1B*), 11q22 (*ATM*), 13q14 (*DLEU1*), and 17p13 (*TP53*) and a break‐apart probe for the region 14q32 (*IGH*) were applied. If results showed a *IGH* split, reflex testing with three *IGH* translocation probes (t(4;14)(p16;q32) [*FGFR3*/*IGH*], t(11;14)(q13;q32) [*CCND1*/*IGH*], and t(14;16)(q32;q23) [*IGH*/*MAF*]) was performed. The probe targeting 1p was changed in August 2016 from 1p36 (D1S2795, D1S253) to 1p32 (*CDKN2C*). In a subset of patients, HRD status was evaluated with the locus‐specific probes for the chromosomal regions 5p15 (D5S1518E/D5S1976), 9q22 (D9S1783), and 15q22 (*SMAD6*). Hybridization was performed according to the manufacturer's instructions (Kreatech, Amsterdam, Netherlands; MetaSystems, Altlussheim, Germany; Vysis/Abbott, Downers Grove, IL, USA). The thresholds in unsorted samples were set at 5% for gains and translocations and at 10% for deletions. For plasma cell‐enriched samples the thresholds recommended by the European Myeloma Network were used (structural abnormalities: 10%, numerical abnormalities: 20%).^13^ If subsequent patient samples were available, retrospective analyses were performed solely with the result of the first obtained sample, unless otherwise stated. In the text of the article, a chromosomal gain without corresponding CN specification (eg, gain(1q)) is defined as three or more copies. The HRD was defined by a gain of any two of the chromosomal regions 5p15, 9q22, or 15q22.^24^ Tetraploidy was predicted if three or more chromosomal regions had four or more copies detected with the standard FISH panel (1p36 or 1p32, 1q21, 11q22, 13q14, 14q32, and 17p13). Cytogenetic cancer clonal fraction of a particular aberration was calculated by dividing the number of affected cells by the number of aberrant cells with the largest detected aberration in the sample. Aberrations were classified as clonal or subclonal using 2/3 as cutoff.

### 2.3 Statistical analyses

Time to next treatment (TTNT) was defined as time from treatment start to the date of starting second‐line therapy, death from any cause, or the last follow‐up. A new line of therapy was defined according to current guidelines.^25^ The OS was calculated from treatment start until death from any cause or the last follow‐up. Both TTNT and OS were estimated with the Kaplan‐Meier method. Statistical differences between the survival curves were analyzed using the log‐rank test. Univariate and multivariate analyses were performed with Cox regression models. The multivariate Cox regression models were adjusted for age, gender, induction therapy, beta‐2 microglobulin (B2M), and high‐risk CA. Additional CA with complete data and *P* values < .1 in the univariate Cox regression analyses were included in the multivariate Cox models. Continuous variables were analyzed using the Wilcoxon rank‐sum test. Association between categorical variables was examined with Fisher's exact test, and *P* values were adjusted for multiple testing using the Benjamini‐Hochberg method. The two‐sided significance level was set at *P* value < .05. All computational analyses were performed using R version 3.6.0 (www.r-project.org/). The R packages included ggplot2, survival, and survminer.

## 3 RESULTS

### 3.1 Patient characteristics

Patient characteristics are shown in Table S1. Median age of the 794 myeloma patients was 70 years (range, 34‐93 years). Most of the patients (>95%) were treated by an induction with immunomodulatory drugs (IMiD) and/or proteasome inhibitors (PI), 44% underwent a front‐line autologous stem cell transplantation (ASCT), and 39% received maintenance therapy.

### 3.2 Cytogenetic landscape

**FIGURE 1:**
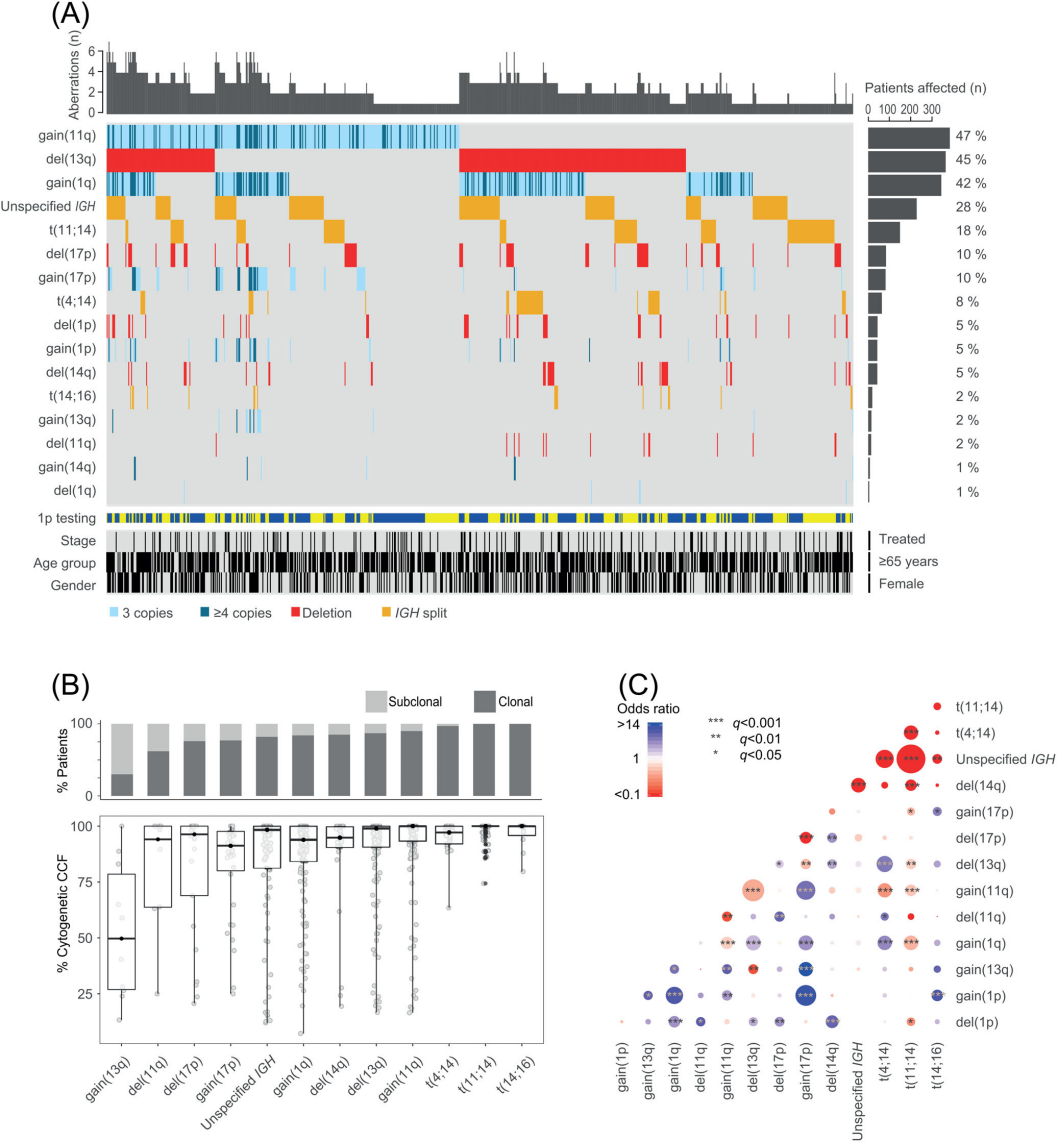
Cytogenetic landscape. (A) Co‐segregation of chromosomal abnormalities in 794 myeloma patients detected with FISH probes. Samples were annotated for 1p testing (1p36: blue, 1p32: yellow), stage (treated vs untreated), age group (≥65 years vs <65 years), and gender (female vs male); (B) Percentage of cases in which a cytogenetic aberration is found to be subclonal or clonal is shown across the patient samples subjected to CD138+ plasma cell enrichment (n = 344). Abnormalities with a frequency of ≥2% in the cohort are shown in the panel. The boxplot showing the cytogenetic cancer clonal fraction (CCF) of the chromosomal abnormalities shows the median (thick black horizontal line) and at the vertical extremities of the boxes the 25th and 75th percentiles. Whiskers' ends represent minimum and maximum values; (C) Pairwise associations between the cytogenetic aberrations present in ≥2% of 794 myeloma patients. Associations are defined with Fisher's exact test; blue color indicates a positive association, whereas red color indicates a negative association. Adjustment for multiple testing was done using the Benjamini‐Hochberg method and the size of the circle depicts the significance of the q value. Abnormalities of 1p (ie, gains or deletions) relate to either 1p36 or 1p32. Unspecified *IGH* indicates at least one unspecified *IGH* abnormality

An overview of the detected CA in the whole cohort is given in Figure 1A. Most common amplified regions (defined as regions with four or more copies) were observed at 1q21 and at 11q22 in 15% (119/794) and 11% (89/794) of the patients, respectively (Table S2). In untreated patients, 3% of the cases showed multiple amplifications suggesting tetraploidy; the number of predicted tetraploid cases increased to 7% in treated patients (Figures S1 and S2). Analysis of the cytogenetic clonal cancer fraction confirmed the oncogenic model of primary and secondary CA: *IGH* translocations t(4;14), t(11;14), and t(14;16) considered as primary events, were almost exclusively clonal and known secondary events such as gain(1q) or del(17p) were more often found to be subclonal (Figure 1B). Pairwise associations confirmed the cytogenetic subgroups t(4;14) and t(11;14) as mutually exclusive (Figure 1C). The analysis also showed a negative association between gain(11q) and both t(4;14) and t(11;14) cases. Translocation t(4;14) was associated with del(13q) and gain(1q), both of which are known to be linked to this subgroup.^10^ Deletions (eg, del(14q), del(13q), and del(17p)) were associated with each other.

### 3.3 Associations of subgroups

Based on our finding that the frequent gain(11q) was negatively associated with primary genetic events (ie, t(4;14) and t(11;14)), we introduced for subgroup analysis a subgroup that was defined by clonal gain(11q) and lack of primary *IGH* translocations (CG11q). Previous studies showed that within the HRD subgroup two clusters can be distinguished according to the presence of a chromosome 11 gain.^10^, ^17^ A substantial part of chromosome 11 gains is also found in the t(11;14) subgroup.^10^, ^26^ Therefore, cases with both gain(11q) and lack of primary *IGH* translocations (eg, t(11;14)) might often belong to the HRD cluster that harbors a chromosome 11 gain (HRD11+). We focused on clonal gain(11q), because as an early event the aberration might have a primary impact on pathogenesis. Using Fisher's exact test we investigated the correlation between five subgroups (ie, t(4;14), t(11;14), t(14;16), CG11q, and a group with the remaining cases) and CA, including amplifications, immunophenotypic findings, and clinical features (Tables S2 and S3). As expected, the CG11q subgroup was characterized by an association with HRD (*P* < .001), and, as reported in studies for HRD,^27^, ^28^ the subgroup was associated with IgG (*P* < .05) and correlated with antigenic coexpression of CD56 and CD117 (CG11q: 50% vs non‐CG11q: 11%, *P* < .001; Tables 1, S2, and S3). About 20% of the CG11q cases showed an amplification of 11q22 (four or more copies). Furthermore, the t(14;16) subgroup was associated with tetraploidy (*P* < .001), plasma cell leukemia (PCL; *P* < .05) and lack of CD56 expression (*P* < .05) (Tables S2 and S3). The distribution of secondary high‐risk CA showed that del(17p) was relatively evenly distributed across the different subgroups (6%‐14% of the cases), while gain(1q) was enriched (*P* < .001) in t(4;14) cases (71%) and in the remaining cases (56%). These two groups and t(14;16) cases were also associated (*P* < .05) with four or more copies of 1q21 in 30%, 20%, and 53% of the cases, respectively. On the other hand, gain(1q) was negatively associated (*P* < .001) with CG11q and t(11;14) subgroups and present in 29% and 25% of the cases, respectively. Interestingly, del(1p32) was detected in all analyzed subgroups (2%‐18% of the cases), whereas del(1p36) was exclusively found in the subgroup with the remaining cases (6% of the cases). Note, del(1p32) was associated with adverse markers such as gain(1q) (*P* < .05), four or more copies of 1q21 (*P* < .001), and del(17p) (*P* < .05), while del(1p36) had significant associations (*P* < .05) with del(13q) and del(14q) (Table S4). Other than gender, immunoglobulin types, and PCL, no clinical feature or treatment schedule was associated with the defined subgroups (Table S3).

**TABLE 1:** Associations between CG11q^a^ subgroup and clinical findings

	All cases (n = 794)		CG11q^a^ cases (n = 271)		Non‐CG11q^a^ cases (n = 523)	
Variable	n/N		n/N		n/N	
Female gender	373/794	47%	**106/271**	**39%** *	**267/523**	**51%** *
Age (≥65 y)^b^	536/794	68%	187/271	69%	349/523	67%
BM plasma cell (≥60%)^b^	175/754	23%	50/253	20%	125/501	25%
Samples from pretreated patients	135/794	17%	48/271	18%	87/523	17%
MACS enriched samples	344/794	43%	118/271	44%	226/523	43%
IgG	203/365	56%	**84/127**	**66%** *	**119/238**	**50%** *
IgA	81/365	22%	28/127	22%	53/238	22%
Light chain only	76/365	21%	**15/127**	**12%** *	**61/238**	**26%** *
Other^c^	4/365	1%	0/127	0%	4/238	2%
Kappa	239/370	65%	89/125	71%	150/245	61%
Lambda	127/370	34%	36/125	29%	91/245	37%
LDH (increased)	61/264	23%	15/87	17%	46/177	26%
Creatinine (≥2 mg/dL)	46/273	17%	15/92	16%	31/181	17%
B2M (≥5.5 mg/L)	74/247	30%	19/80	24%	55/167	33%
Hemoglobin (<10 g/dL)	85/247	34%	25/79	32%	60/168	36%
Platelets (<150 x 10^9^/L)	57/249	23%	17/79	22%	40/170	24%
Calcium (≥2.75 mmol/L)	17/260	7%	5/88	6%	12/172	7%
Serum involved/uninvolved FLC ratio (≥100)	91/222	41%	31/76	41%	60/146	41%
ISS I	66/236	28%	26/77	34%	40/159	25%
ISS II	96/236	41%	31/77	40%	65/159	41%
ISS III	74/236	31%	20/77	26%	54/159	34%
R‐ISS I	42/219	19%	18/69	26%	24/150	16%
R‐ISS II	144/219	66%	46/69	67%	98/150	65%
R‐ISS III	33/219	15%	5/69	7%	28/150	19%
PI‐based induction	123/311	40%	45/107	42%	78/204	38%
IMiD‐based induction	14/311	5%	7/107	7%	7/204	3%
PI‐based and IMiD‐based induction	165/311	53%	52/107	49%	113/204	55%
Other^d^	9/311	3%	3/107	3%	6/204	3%
Maintenance	111/287	39%	40/101	40%	71/186	38%
Front‐line ASCT	135/307	44%	51/104	49%	84/203	41%
EMM^e^	52/328	16%	15/111	14%	37/217	17%
PCL^f^	24/328	7%	3/111	3%	21/217	10%
AL	14/323	4%	4/110	4%	10/213	5%

*Note*: Clinical features at diagnosis, unless otherwise indicated. All statistically significant values are in bold.

Abbreviations: AL, Amyloidosis; ASCT, autologous stem cell transplantation; B2M, beta‐2 microglobulin; BM, bone marrow; FLC, free light chain; IMiD, immunomodulatory drugs; ISS, International Staging System; LDH, lactate dehydrogenase; MACS, magnetic‐activated cell sorting; PI, proteasome inhibitors; R‐ISS, revised International Staging System.

^a^ Subgroup clonal gain(11q) (CG11q) was defined as the presence of clonal gain(11q) and the absence of t(4;14), t(11;14), and t(14;16).

^b^ Clinical feature the time of first sampling.

^c^ This category includes IgD, IgM, and nonsecretory MM.

^d^ This category includes alkylating agents and monoclonal antibodies.

^e^ Extramedullary myeloma (EMM) was present at diagnosis or developed during disease course and was defined as plasma cell infiltration of the soft tissue (extramedullary extraosseous and/or extramedullary‐bone related).

^f^ Plasma cell leukemia (PCL) was present at diagnosis or developed during disease course.

* *P* < .05, two‐sided Fisher's exact test. For multiple testing *P* values were adjusted with the Benjamini‐Hochberg method.

### 3.4 Associations with female gender

The subgroups t(14;16) and CG11q were associated (*P* < .05) with female and male gender, respectively (Figure S3A, Tables 1 and S3). To analyze whether further gender differences were present in our cohort, we studied associations between gender and all CA and clinical characteristics. Additionally, female patients displayed a significantly higher frequency of del(13q) than did male patients (*P* < .001; Figure S3A, Table S4). This could not be explained by the observation that specific cytogenetic subgroups that co‐occur with del(13q) are more prevalent in female patients (Figure S3B). Furthermore, we found a significantly larger number of cytogenetic aberrations in female patients (*P* < .05; Figure S3C). Female gender was also positively associated (*P* < .05) with light chain only myeloma and an increased serum involved/uninvolved free light chain (FLC) ratio (≥100) (Table S4).

### 3.5 Survival of the whole cohort

During a median follow‐up of 2.7 years (range, 0‐14.5), 211 TTNT events and 137 deaths were observed in 299 patients. Median TTNT and OS were 2.0 and 5.8 years, respectively. Survival analysis of all patients with available follow‐up data showed that TTNT and OS were significantly different in the defined subgroups (Figure S4). Univariate Cox analyses for TTNT and OS of the whole cohort were performed using cytogenetic, immunophenotypic, and clinical features. The results are shown in Table S5. Several parameters were associated with shorter TTNT and OS: gain(1q) (both three and four or more copies), del(13q), del(17p), B2M of 5.5 mg/L or higher, hemoglobin less than 10 g/dL, creatinine of 2 mg/dL or higher, platelets less than 150 x 10^9^/L, calcium of 2.75 mmol/L or greater, serum involved/uninvolved FLC ratio (≥100), International Staging System stage III (ISS III),^29^ revised ISS (R‐ISS) III,^16^ high‐risk CA,^16^ double‐hit and triple‐hit (co‐occurrence of two or three adverse lesions, respectively),^17^, ^19^, ^20^ four or more copies of 1q21 plus ISS III (defined as Double‐Hit myeloma),^18^ and PI‐based induction. The parameters ISS I, R‐ISS I, front‐line ASCT, and CD27 were associated with longer TTNT and better OS. Median TTNT was 1.3 vs 1.7 vs 2.8 years (log‐rank *P* < .001) and median OS was 3.1 vs 4.1 vs 6.9 years (log‐rank *P* < .001) for four or more copies, three copies, and two or fewer copies of 1q21, respectively (Figures S5A,B). In the multivariate Cox analysis, four or more copies of 1q21 (but not three copies), high‐risk CA and B2M of 5.5 mg/L or higher were independent adverse prognostic factors for TTNT and OS (Figures S5C,D). Some features were shown to be associated with survival in univariate analysis, but not included in the multivariate analysis because of their correlation with B2M and/or their incompleteness of data (Tables S4 and S5; Figure S6). In addition, high‐risk groups that comprised gain(1q) (eg, double‐hit) as well as del(17p), t(4;14), and t(14;16) alone, which together defined the already included high‐risk CA, were not used as factors. Patients with gain(1q) who underwent front‐line ASCT had a longer TTNT (log‐rank *P* = .016) and OS (log‐rank *P* = .004) than did patients with gain(1q) who did not receive front‐line ASCT (Figure S7). However, the significance was lost when cases with very short OS (less than six months) were excluded from the analysis. Furthermore, PI‐based and IMiD‐based induction and maintenance regimens were not associated with a statistically significant better outcome in patients with gain(1q).

### 3.6 Impact of gain(1q) on survival of patients in the CG11q subgroup

**FIGURE 2:**
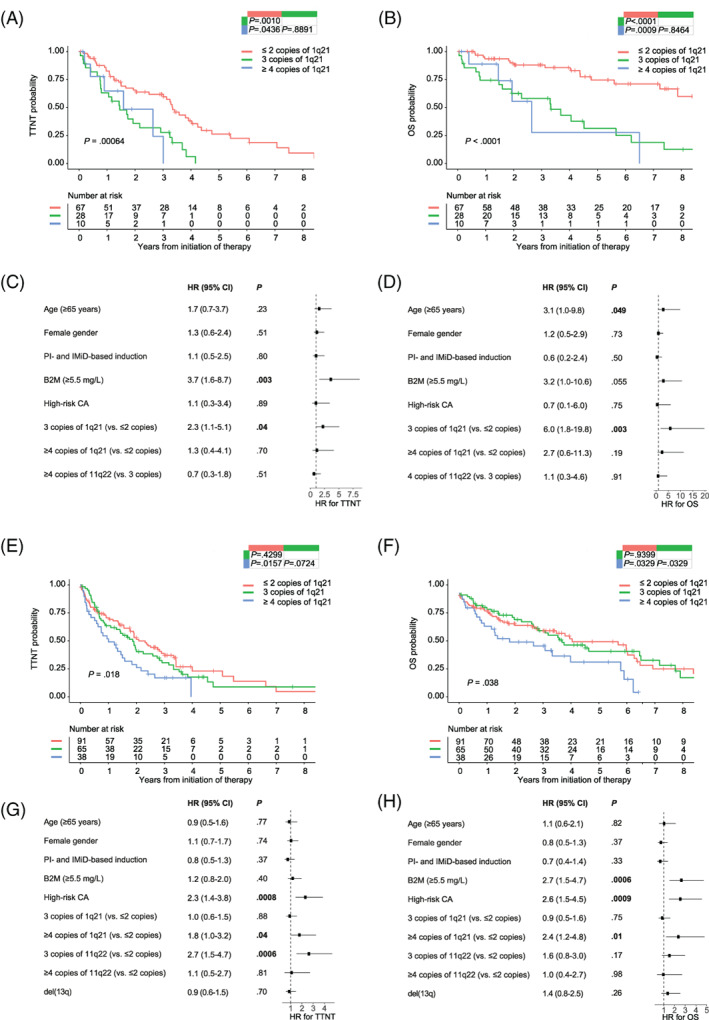
Survival of patients with gain(1q) in the CG11q subgroup and in the non‐CG11q subgroup. Kaplan–Meier curves for (A), time to next treatment (TTNT) and (B), overall survival (OS) stratified according to 1q21 copy number (CN) status in the subgroup clonal gain(11q) (CG11q); forest plots show results of the multivariate analysis for (C), TTNT and (D), OS in the CG11q subgroup. Kaplan–Meier curves for (E), TTNT and (F), OS stratified according to 1q21 CN status in the non‐CG11q cases; forest plots show results of the multivariate analysis for (G), TTNT and (H), OS in the non‐CG11Q cases. Statistical significance of the difference between curves was tested using the log‐rank test; *P* values of pairwise comparisons are shown in the upper right table inside the figure. Hazard ratio on the x‐axis of the forest plots, values <1 are associated with better prognosis, values >1 are associated with poorer prognosis. High‐risk chromosomal abnormalities (CA) were defined as del(17p), t(4;14), and t(14;16). B2M, beta‐2 microglobulin; CI, confidence interval; IMiD, immunomodulatory drugs; PI, proteasome inhibitors

Next, we separated the CG11q subgroup from the remaining cases to perform CG11q subgroup‐specific Cox univariate analysis. Age ≥ 65 years, gain(1q) (both three and four or more copies), creatinine of 2 mg/dL or higher, B2M of 5.5 mg/L or higher, ISS III, double‐hit, four or more copies of 1q21 plus ISS III, and PI‐based induction were associated with a negative impact on both TTNT and OS (Table S6). Front‐line ASCT was associated with longer TTNT and better OS. Median TTNT was 1.6 vs 1.5 vs 3.3 years (log‐rank *P* < .001) and median OS was 2.6 vs 3.3 vs 9.6 years (log‐rank *P* < .001) for four or more copies, three copies, and two or fewer copies of 1q21, respectively (Figure 2A,B). In the multivariate analysis, gain(1q) with three copies was the only parameter that retained its adverse prognostic value for both TTNT and OS (Figure 2C,D).

### 3.7 Impact of gain(1q) on survival of patients in the non‐CG11q cohort

In the univariate Cox analysis of non‐CG11q cases the following parameters were associated with adverse TTNT and OS: four or more copies of 1q21, three copies of 11q22, del(13q), del(17p), high‐risk CA, double‐hit, triple‐hit, four or more copies of 1q21 plus ISS III, B2M of 5.5 mg/L or higher, serum involved/uninvolved FLC ratio (≥100), ISS III, R‐ISS III, and PI‐based induction. Both R‐ISS I and CD27 were associated with better outcome (Table S7). Median TTNT was 1.0 vs 1.9 vs 2.1 years (log‐rank *P* = .018) and median OS was 3.1 vs 4.6 vs 6.0 years (log‐rank *P* = .038) for four or more copies, three copies, and two or fewer copies of 1q21, respectively (Figure 2E,F). The survival curves between three copies and two or fewer copies were not significantly different. Gain(1q) with four or more copies and high‐risk CA remained adverse prognostic factors in the multivariate analysis for TTNT and OS (Figure 2G,H). Of note, neither three copies of 1q21 nor four or more copies of 1q21 had a significant impact on survival in the t(4;14) and t(11;14) subgroups alone (Figure S8A‐D). It seems that the observable poor impact of four or more copies of 1q21 in the non‐CG11q cases is mainly driven by the remaining cases (Figure S8E,F).

### 3.8 Associations of high‐risk myeloma

**FIGURE 3:**
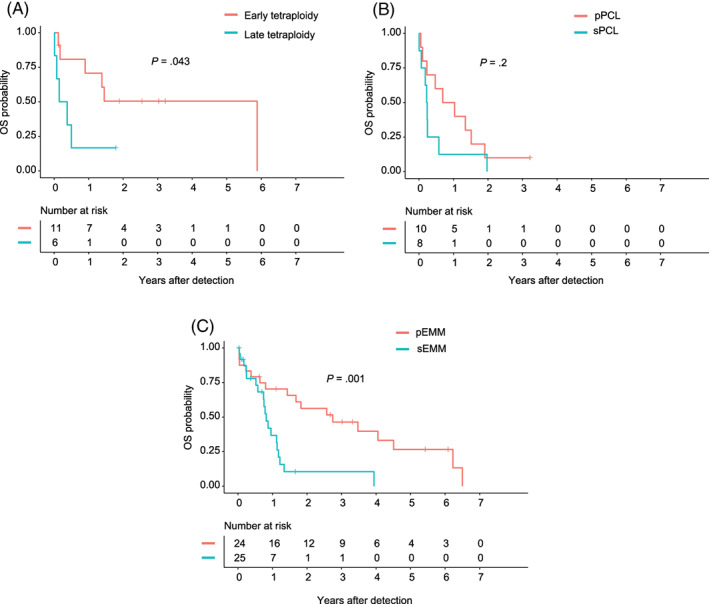
(A) Overall survival (OS) after detection of early tetraploidy vs late tetraploidy, (B) OS after detection of primary plasma cell leukemia (pPCL) and secondary PCL (sPCL), (C) OS after detection of primary extramedullary multiple myeloma (pEMM) and secondary EMM (sEMM) is shown. The log‐rank test was used to perform group comparisons

In our cohort, the number of patients with multiple amplified regions increased during disease course, mirroring clonal evolution. We studied cytogenetic and clinical features of patients with tetraploidy and patients having advanced disease manifestations such as extramedullary multiple myeloma (EMM) and plasma cell leukemia (PCL) (Tables S8 and S9). Patients with tetraploidy were divided into a group with early manifestation of tetraploidy and another group that acquired tetraploidy during the course of disease. Early tetraploidy was significantly associated (*P* < .001) with greater BM infiltration (≥60% infiltration) and the t(14;16) subtype. A double‐hit myeloma as defined by Walker et al^18^ (≥4 CN gain(1q) plus ISS III) was found in 60% (6/10) of patients with early tetraploidy (*P* < .001). Primary *IGH* translocations (ie, t(4;14), t(11;14), and t(14;16)) and high‐risk CA were common cytogenetic features of both tetraploidy (*P* < .05) and PCL (*P* < .05 and *P* < .001, respectively). Baseline characteristics such as del(17p), hemoglobin less than 10 g/dL, and platelets less than 150 x 10^9^/L were associated with primary PCL (pPCL) in 38% (*P* < .05), 80% (*P* < .05), and 89% (*P* < .001) of cases, respectively. Secondary EMM (sEMM) was associated with patients who received three or more lines of therapy (76% of the patients; *P* < .001) and acquired a tetraploid clone (23% of the patients; *P* < .05) during their course of disease. No other specific association with clinics or cytogenetics (eg, subgroup) was observed for EMM. Median time between therapy initiation and diagnosis of secondary PCL (sPCL), sEMM, and late tetraploidy was 1.6, 1.8, and 2.8 years, respectively. Primary and sPCL, sEMM, and late tetraploidy were all associated with very poor survival after detection (less than one year), whereas pEMM and early tetraploidy were associated with a relatively better outcome (Figure 3A‐C).

## 4 DISCUSSION

Our results highlight the importance of defining cytogenetic subgroups in MM as this has an impact on the course of the clinical disease. It may be of particular importance to integrate a more advanced subgrouping into future clinical trials to avoid over‐treatment or under‐treatment of defined (cytogenetic) risk groups. This statement is underscored by our observation that the cytogenetic subgroups defined here significantly differed with respect to CA, antigen expression, clinical features, and prognosis. As reported earlier,^18^, ^21^, ^22^, ^23^, ^30^ four or more copies of 1q21 were associated with a more negative impact on survival than were three copies of 1q21 in the whole cohort. However, the prognosis associated with a different number of copies of gain(1q) was dependent on the subgroup. The CG11q subgroup (~35% of all patients), defined by clonal gain(11q) without primary *IGH* translocations, was characterized by an association with HRD, coexpression of CD56 and CD117 (*KIT*), and IgG subtype. The majority of these patients most probably belong to the distinct HRD group with chromosome 11 gain.^10^, ^17^ The CG11q patients had a long median OS of over nine years in the absence of gain(1q); when in about one‐third of the cases a concomitant gain(1q) was present, the favorable prognosis was abrogated, and the median OS was less than four years for patients with three or more copies of 1q21. The strong association between CD56 and CD117 coexpression and CG11q cases was irrespective of the gain(1q) CN status (Table S4), indicating that gain(1q) does not impact the expression of these antigens, which was previously associated with good prognosis (CD117)^31^ and dependence on the BM microenvironment (CD56).^32^ In contrast to the CG11q subgroup, the negative impact of gain(1q) was less pronounced in the non‐CG11q cohort and restricted to four or more copies of 1q21. Amplifications of 1q21 (four or more copies) are known to be accompanied by high‐risk states.^33^ In line with this, we observed an association between four or more copies of 1q21 plus ISS III (double‐hit patients)^18^ and tetraploidy, which has been correlated with genomic instability, advanced disease, and poor prognosis.^34^, ^35^ The adverse prognosis of CG11q patients harboring gain(1q), already observable with three copies of 1q21, might be linked to an increased expression of both *CCND1* (D1; associated with gain(11q)) and *CCND2* (D2; associated with gain(1q)).^17^, ^36^ This co‐overexpression, initially described by Bergsagel et al in the translocation/cyclin D (TC) classification as a feature of a distinct GEP‐based subgroup (D1+D2), was recently demonstrated at the RNA and DNA level in HRD patients in the Myeloma IX and XI trials.^17^, ^36^ The presence of other (associated) genetic lesions (eg, *MYC* rearrangements) in CG11q patients with gain(1q) may also play a role in the observed adverse phenotype.^37^ We analyzed several high‐risk groups with cytogenetic markers such as high‐risk CA (ie, del(17p), t(4;14), and t(14;16)), R‐ISS III, double‐hit as well as triple‐hit, and four or more copies of 1q21 plus ISS III, which in the entire group had an incidence of 20%, 15%, 11%, 1%, and 9%, respectively (Table S2). All of these parameters were significantly associated with adverse survival for the entire cohort (Table S5). However, four of five of these high‐risk definitions were associated with specific non‐CG11q cases (ie, t(4;14) and/or t(14;16) subgroups) and therefore might be less appropriate for the identification of high‐risk patients in other subgroups such as CG11q. In our cohort, similar to findings recently reported,^34^ about 30% of the t(14;16) cases had an early tetraploidy, indicating that whole genome‐doubling is a relatively early event in this MM subgroup. Additionally, our data suggest that tetraploidy acquired in a late phase of the disease is associated with a prognosis that is similarly poor as for sPCL and sEMM. Interestingly, we found gender discrepancies for CA consistent with a previous analysis of the MRC Myeloma IX dataset.^38^ Gender discrepancies, which have been observed in other cancers including hematological malignancies, could also comprise molecular lesions.^39^, ^40^ Analyzing the gender discrepancy in more detail in future studies will contribute to our understanding of the pathobiology of MM. This study is limited by the retrospective nature of data collection. As clinical annotation was very comprehensive in ~40% of the patients, a minimal clinical dataset was analyzed in ~60% of the patients, limiting the power especially for associations with small cytogenetic subgroups. Regarding patient samples, there was technical heterogeneity (ie, partly unsorted samples), which might have influenced the sensitivity of secondary CA detection. On the other hand, a minority of patients (<20%) was pretreated at the time of first FISH analysis. This could possibly have increased the number of secondary CA, if the CA were acquired between treatment initiation and first FISH analysis, and may have introduced a bias towards prolonged TTNT and OS for secondary CA (eg, gain(1q)). Proper evaluation of del(1p) as compared to other CA was hampered because of the 1p FISH probe, which was changed from 1p36 to 1p32 over time. While a strength of our analysis was the sensitive CN detection by FISH, further genetic data from CA (eg, HRD, *MYC* rearrangements, and less frequent translocations) and molecular analysis (eg, CN alterations, gene mutations as determined by NGS) would allow better characterization of the subgroups. Further investigations, ideally in the context of prospective studies, are warranted to confirm and comprehensively elucidate our findings. As the frequencies of some cytogenetic subgroups are very low, large cohorts are required to further refine genetic subgrouping for outcome prediction and therapy tailoring in MM clinical trials.

We conclude that cytogenetic subgroups in MM differ in various aspects in our cohort and that evaluation of secondary genetic events on the basis of cytogenetic subgroups might further improve MM risk stratifications. Our data suggest that already three copies of 1q21 are associated with an adverse outcome in patients of the CG11q/HRD11+ subgroup. The 1q21 testing in this subgroup might enable patients to be stratified in a group with adverse prognosis as well as a group with a very favorable outcome. In non‐CG11q patients, four or more copies of 1q21 (but not three copies) were associated with a significant adverse impact on the outcome.

## CONFLICT OF INTEREST

The authors declare no conflicts of interest.

## Supporting information


**File S1.** Supporting information

## Data Availability

The data that support the findings of this study are available from the corresponding authors upon reasonable request.
