# Supplementary material for: The prognostic value of additional copies of 1q21 in multiple myeloma depends on the primary genetic event
Source: Am J Hematol. 2020 Sep 19;95(12):1562–71. doi: 10.1002/ajh.25994 (PMC7756645; doi:10.1002/ajh.25994)
Supplement: Supplementary file 1 — File S1. Supporting information [file AJH-95-1562-s001.docx]

**SUPPORTING INFORMATION**

The prognostic value of additional copies of 1q21 in multiple myeloma depends on the primary genetic event

Maurus Locher^1^, Michael Steurer^2^, Emina Jukic^1^, Markus A. Keller^1^, Friedrich Fresser^1^, Carmen Ruepp^3^, Ewald Wöll^3^, Irmgard Verdorfer^1^, Günther Gastl^2^, Wolfgang Willenbacher^2,3^, Roman Weger^4^, David Nachbaur^2^, Dominik Wolf^2,5^, Eberhard Gunsilius^2^, Johannes Zschocke^1,6^, and Normann Steiner^2,6^

^1^Institute of Human Genetics, Medical University of Innsbruck, Innsbruck, Austria;

^2^Department of Internal Medicine V, Medical University of Innsbruck, Innsbruck, Austria;

^3^St. Vinzenz Krankenhaus Betriebs GmbH, Zams, Austria;

^4^Oncotyrol–Center for Personalized Cancer Medicine, Innsbruck, Austria;

^5^Medical Clinic 3, Oncology, Hematology, Immunoncology and Rheumatology, University Hospital Bonn, Bonn, Germany

^6^Correspondence

**
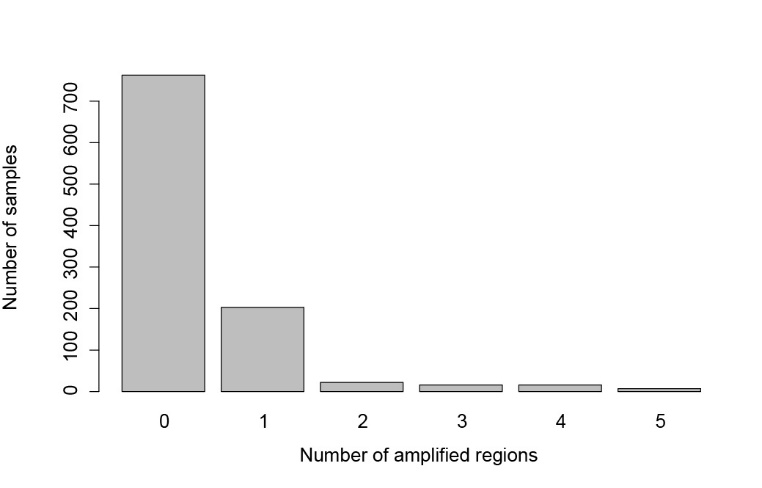
**

**Figure S1. Number of amplified chromosomal regions.** Bar plot displays amplified regions (regions with four or more copies) in 1027 samples from 794 patients.


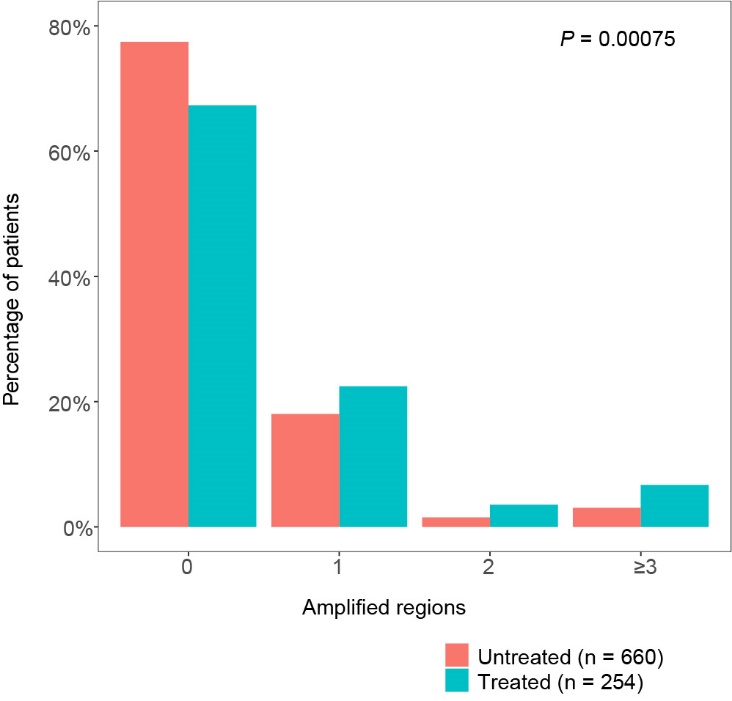


**Figure S2. Number of amplified regions increases during disease course.** Bar graph shows the percentage of patients with amplified loci (four or more copies) analyzed with the standard FISH panel (1p36 or 1p32, 1q21, 11q22, 13q14, 14q32, and 17p13). Patients are grouped in an untreated and/or a treated group. If a patient had samples taken before and after treatment initiation, he was present in both groups. If two or more subsequent samples from a patient were analyzed before treatment start, only the result of the first sample was taken into account. If two or more subsequent samples were analyzed after treatment initiation, only the result of the last obtained sample was taken into account. In the untreated group versus the treated group one, two, or three or more (defined as tetraploidy) amplified regions were present in 18% versus 22%, 2% versus 4%, and 3% versus 7% of the patients, respectively. Statistical significance was assessed using the Wilcoxon rank-sum test.


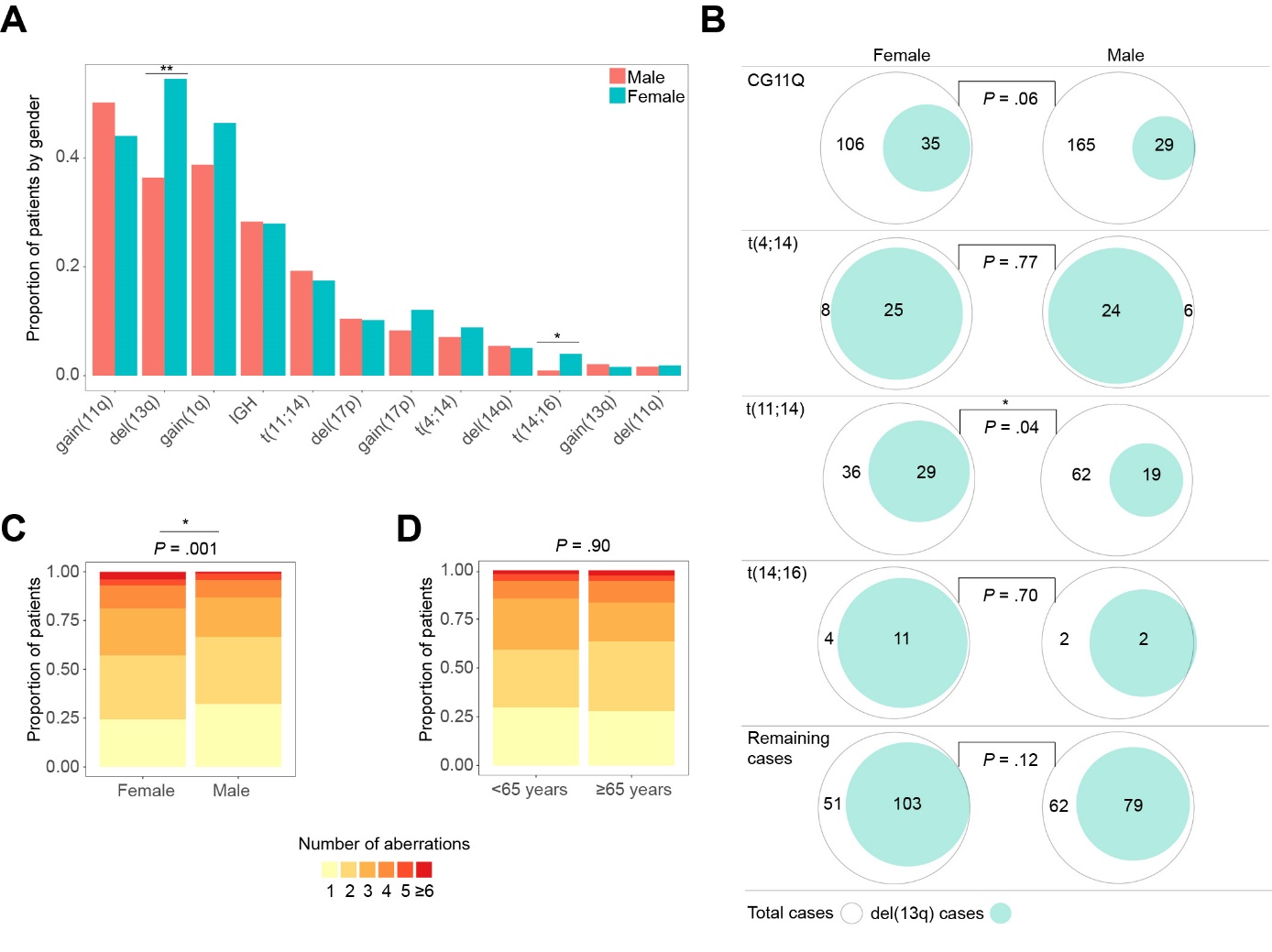


**Figure S3. Gender differences.** A) Bar plot comparing the frequencies of the most common cytogenetic aberrations (present in ≥2% of the total cohort) in male (n = 421) and female (n = 373) patients. Due to the inhomogeneous testing of 1p, del(1p) was not considered in this plot. B) Diagrams show the number of del(13q) in the subgroups clonal gain(11q) (CG11q), t(4;14), t(11;14), t(14;16) and the remaining cases. Empty circle and filled green circle indicate the total number of cases and the number of del(13q) for each subgroup, respectively. Number of aberrations per patient, according to C) gender and D) age (<65 years, n = 258; ≥65 years, n = 536). Co-occurring gain and amplification of a particular chromosomal region were counted once. IGH indicates at least one unspecified IGH abnormality with a split event between 3' IGH and 5' IGH. Statistical significance was assessed using Fisher’s exact test (A-B), with adjustment for multiple testing using the Benjamini-Hochberg method, and the Wilcoxon rank-sum test (C-D) without adjustment for multiple testing. ** *P* < .001; * *P* < .05.


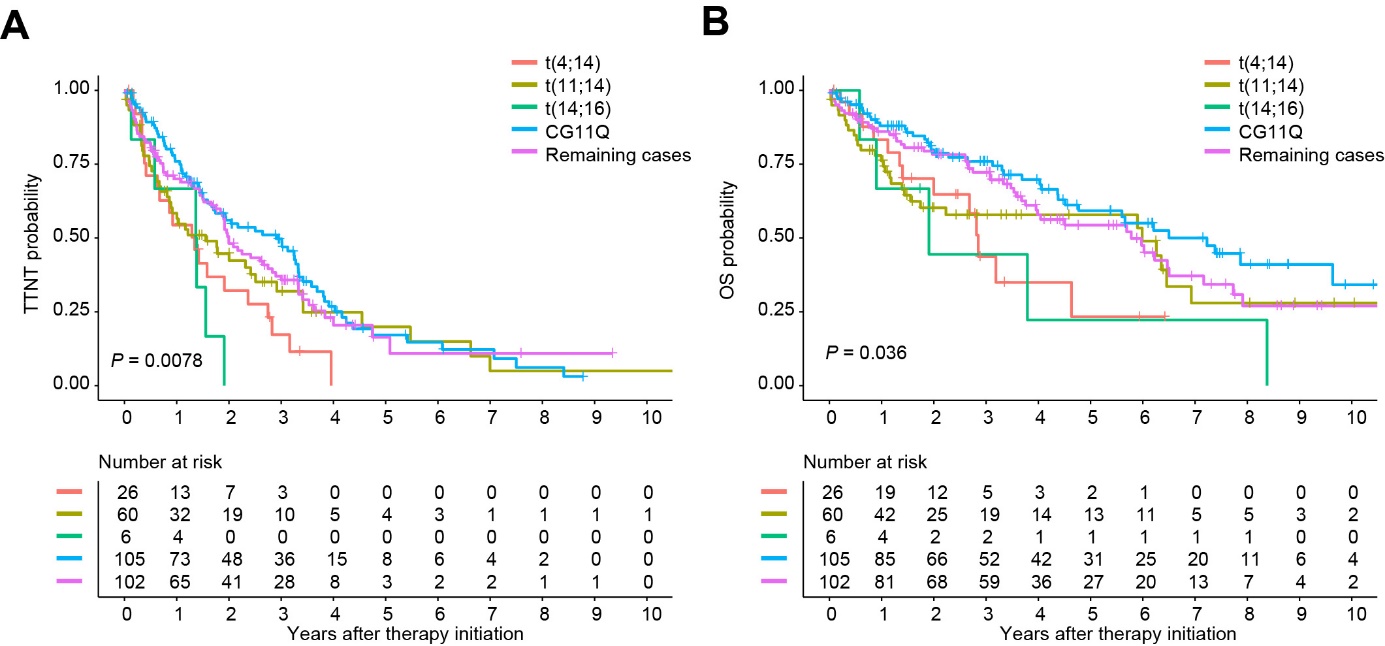


**Figure S4**. **Survival in various cytogenetic subgroups.** Kaplan-Meier curves and log-rank *P* values for A) time to next treatment (TTNT) and B) overall survival (OS) for t(4;14) versus t(11;14) versus t(14;16) versus clonal gain(11q) (CG11q) versus remaining cases.

**
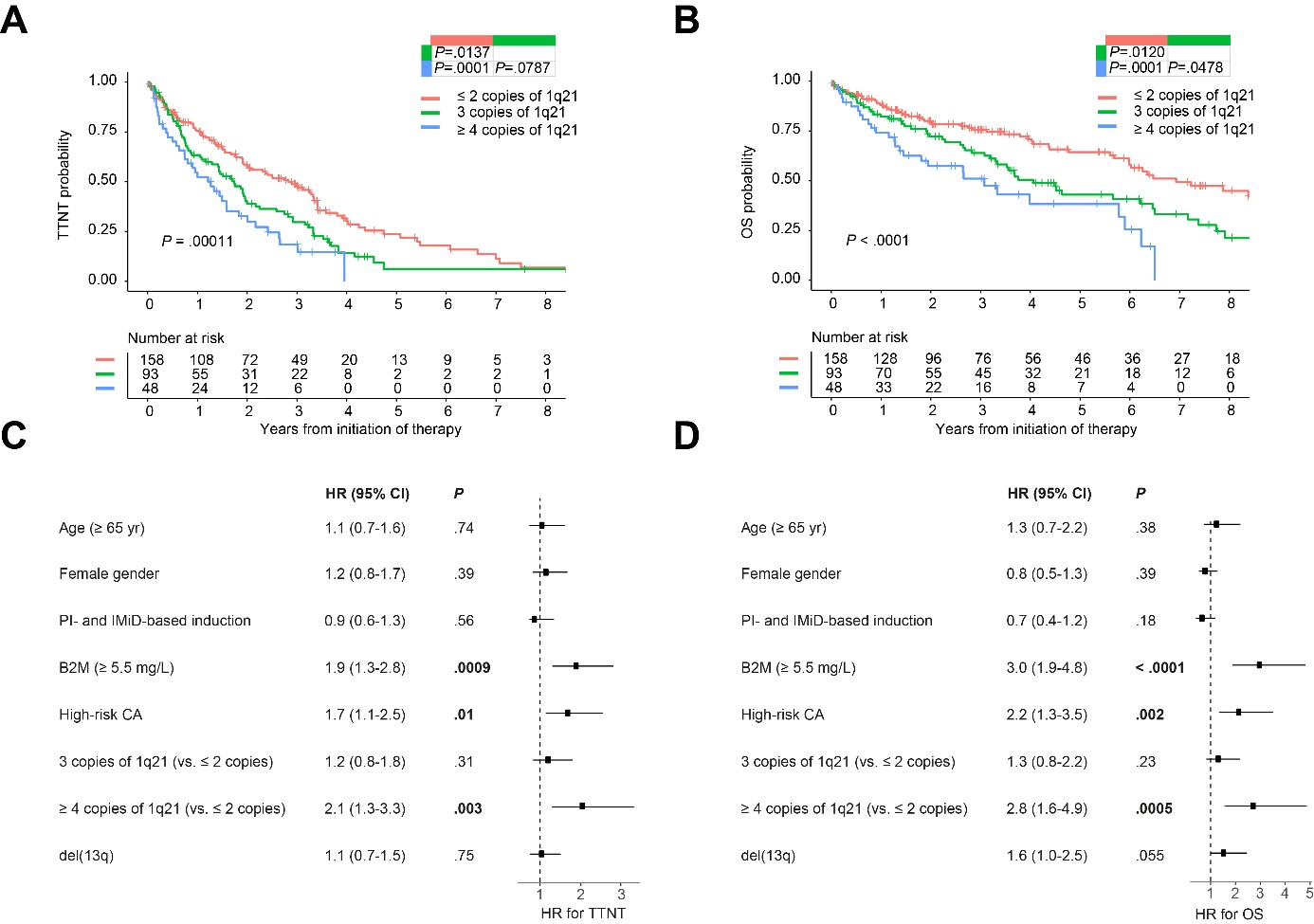
**

**Figure S5.** Survival of patients with gain(1q) and multivariate analysis. Kaplan-Meier curves for (A) time to next treatment (TTNT) and (B) overall survival (OS) stratified according to 1q21 copy number status. Statistical significance of the difference between curves was tested using the log-rank test; *P* values of pairwise comparisons are shown in the upper right table inside the figure. Forest plots show results of the multivariate analysis for (C) TTNT and (D) OS. Hazard ratio on the X axis of the forest plots, values <1 are associated with better prognosis, values >1 are associated with poorer prognosis. High-risk chromosomal abnormalities (CA) were defined as del(17p), t(4;14), and t(14;16). Abbreviations: B2M, beta-2 microglobulin; CI, confidence interval; IMiD, immunomodulatory drugs; PI, proteasome inhibitors.


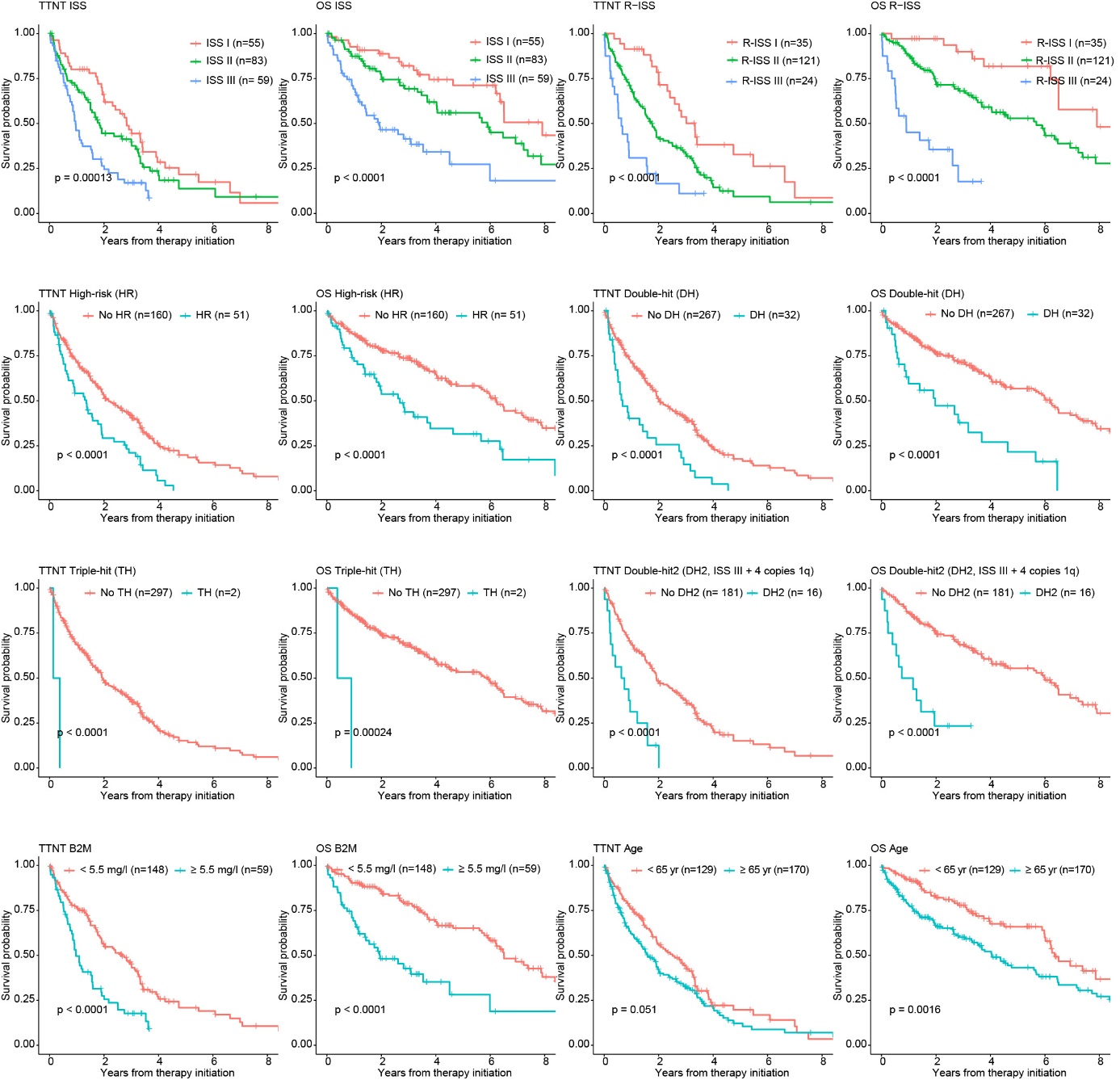


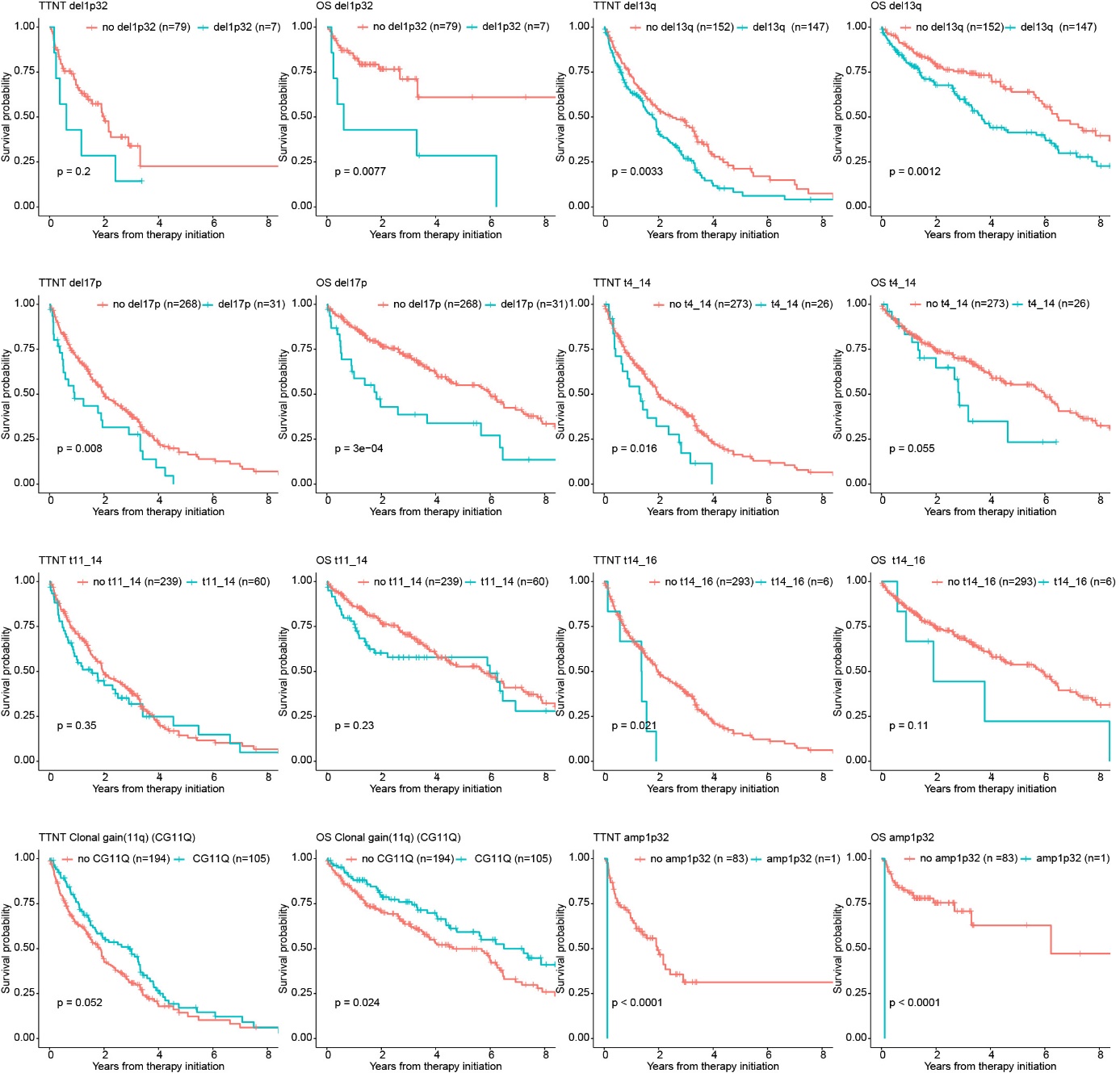


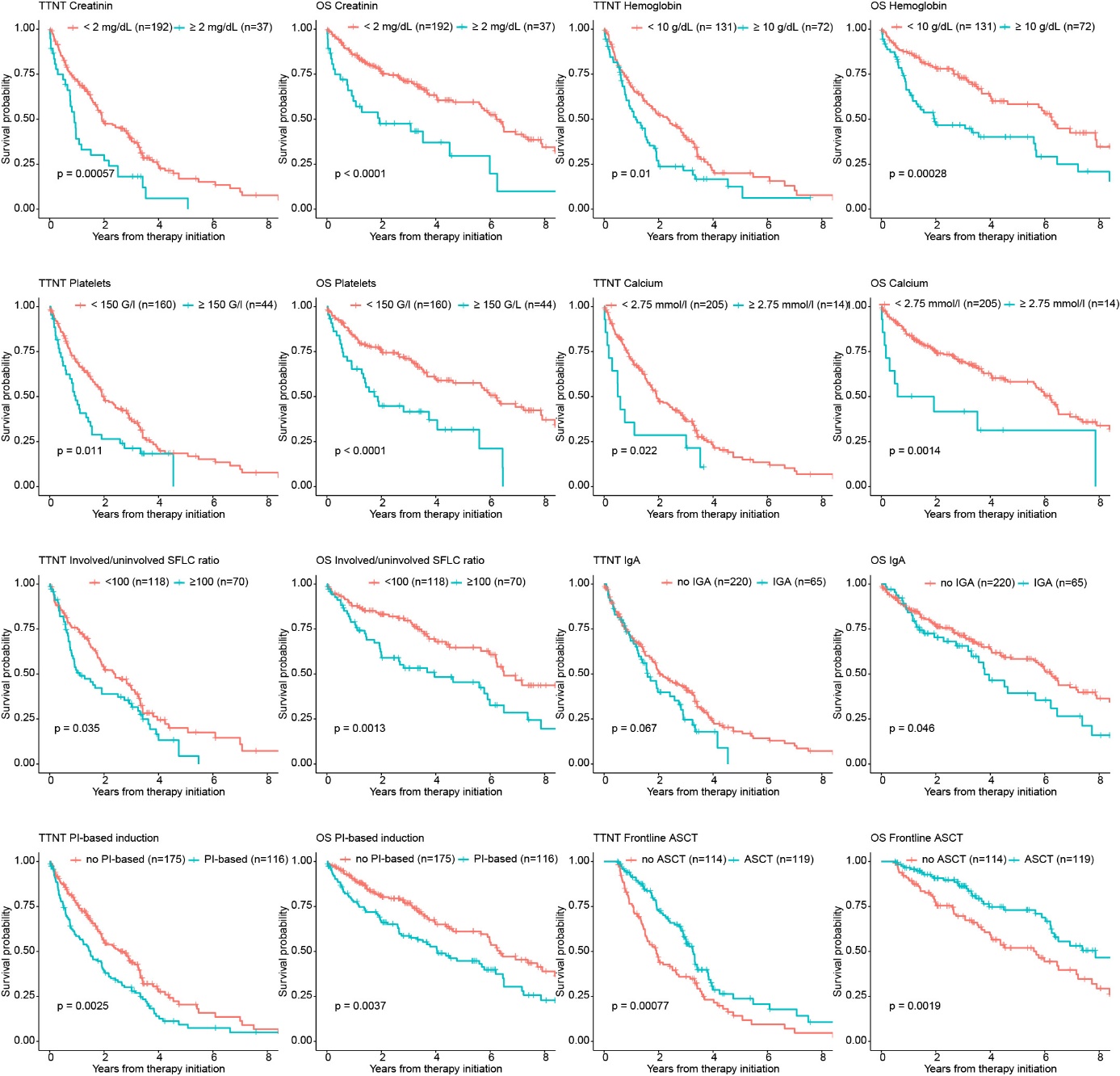


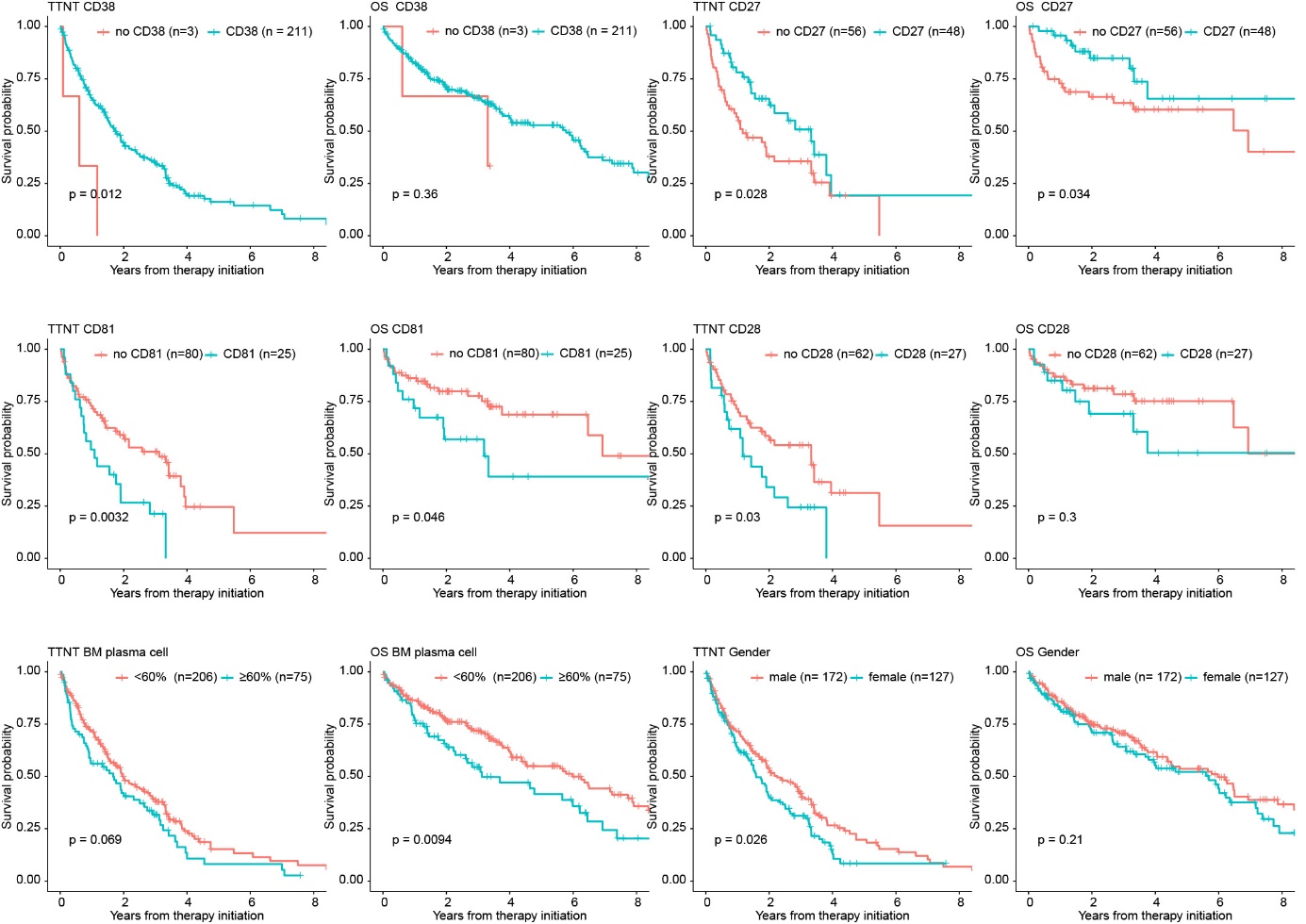


**Figure S6.** **Univariate analysis.** Kaplan-Meier curves and log-rank *P* values for time to next treatment (TTNT) and overall survival (OS) of prognostic factors of the whole cohort identified by univariate Cox analysis (Table S5).


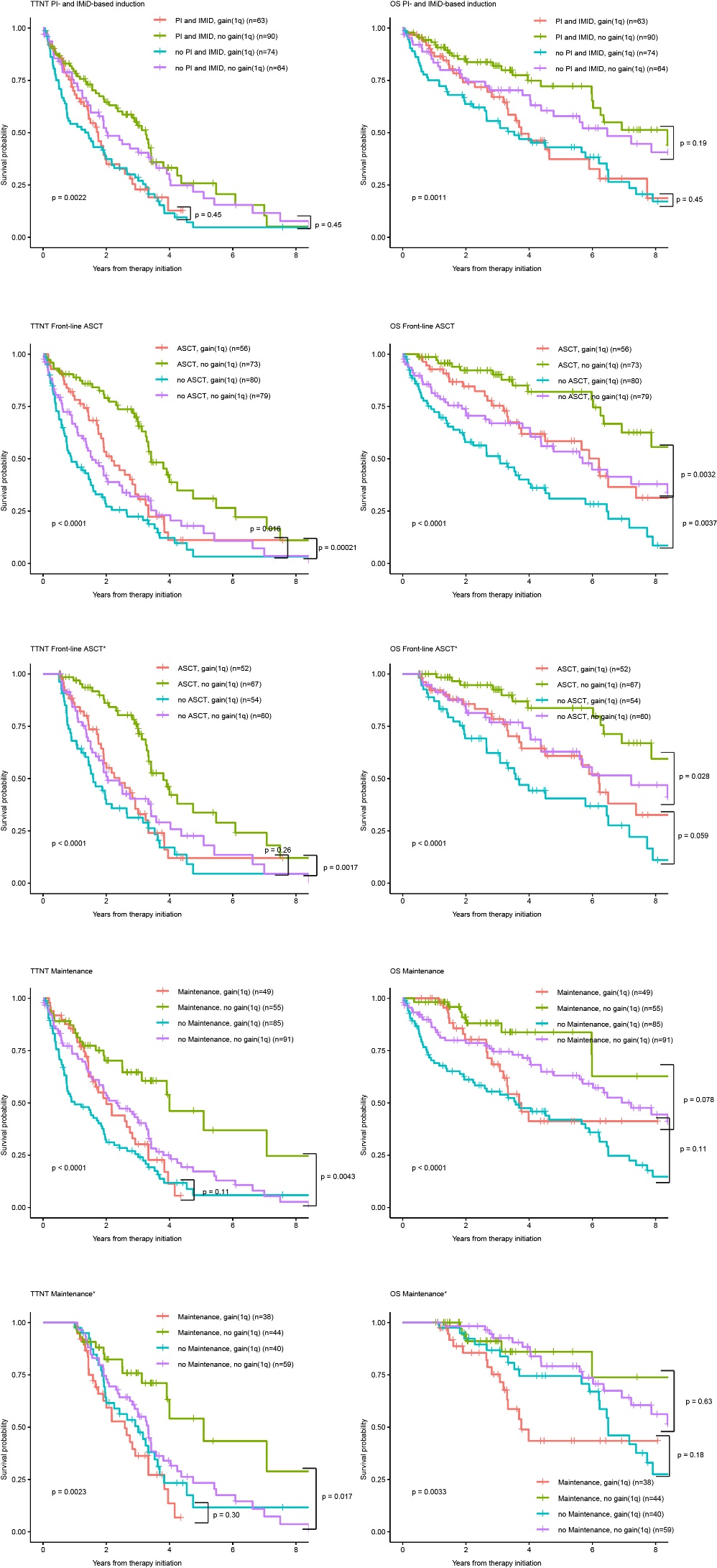


**Figure S7. Impact of gain(1q) on time to next treatment (TTNT) and overall survival (OS) stratified by therapy regimens.** Statistical difference between curves was tested using the log-rank test. An asterisk indicates that only cases with a TTNT or OS of ≥ 6 months (front-line ASCT) or ≥ 12 months (maintenance therapy) were considered; cases with an early treatment change or death could potentially introduce a bias in favor of treatments that are applied later (i.e., ASCT and maintenance). ASCT, autologous stem cell transplantation; IMiD, immunomodulatory drugs; PI, proteasome inhibitors.

**
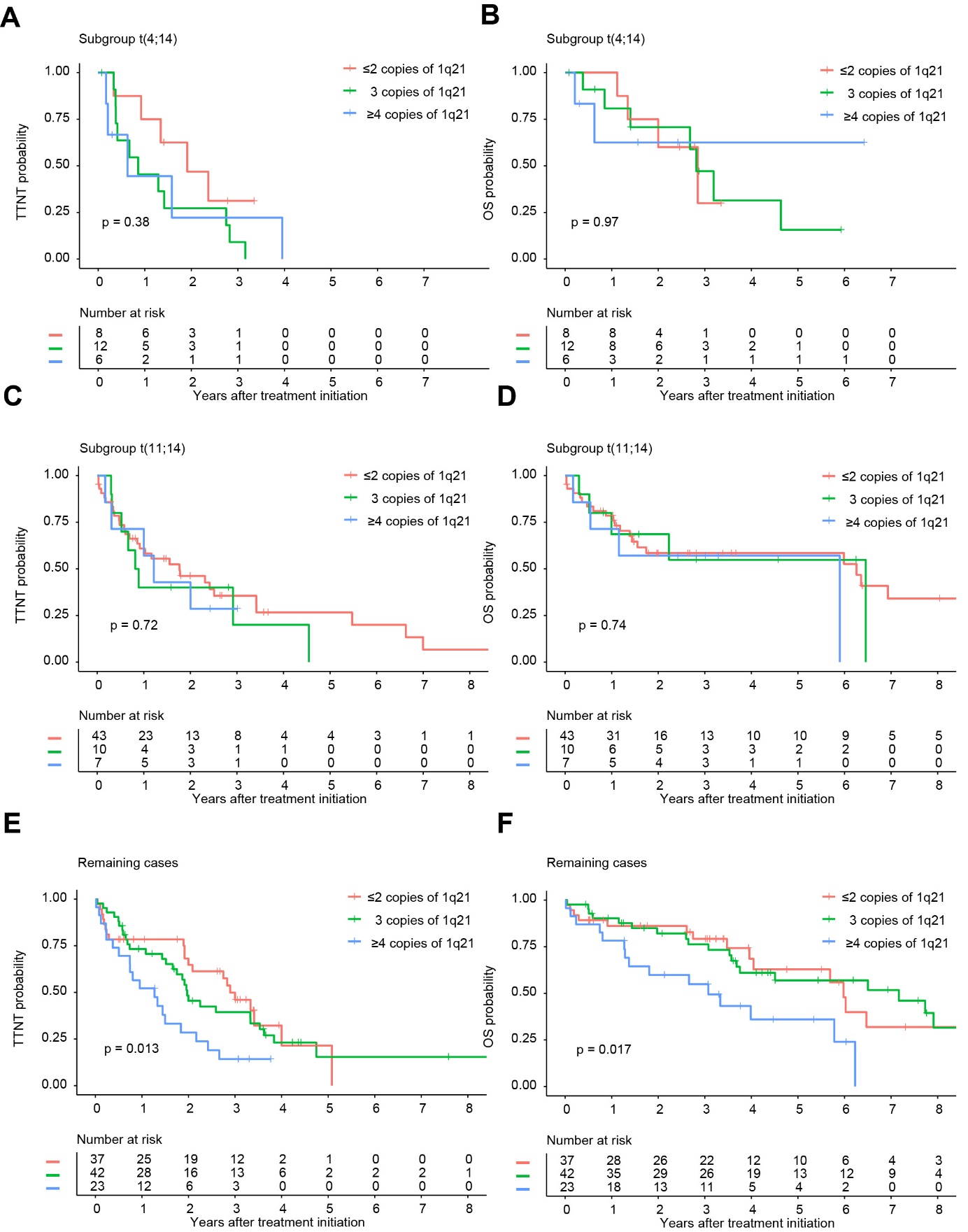
**

**Figure S8. Survival of patients with gain(1q) in the subgroups without clonal gain(11q) (non-CG11q).** Kaplan-Meier curves for time to next treatment (TTNT) (A, C, and E) and overall survival (OS) (B, D, and F) stratified according to 1q21 copy number (CN) status in the subgroups t(4;14) and t(11;14) and in the remaining cases, respectively. Due to the small number, the subgroup t(14;16) was not analyzed. Statistical significance of the difference between curves was tested using the log-rank test.

| **Table S1. Patient characteristics** | | | | | |
| --- | --- | --- | --- | --- | --- |
|  |  | **All patients (N = 794)** | | **Patients with survival data (N = 299)** | |
| **Characteristic** | **Category** | **Value** | **N** | **Value** | **N** |
| Age^a^, yr, median (range) | NA | 70 (34-93) | 794 | 67 (34-91) | 299 |
| LDH, U/L, median (range) | NA | 200 (69-1539) | 264 | 199 (69-981) | 221 |
| Albumin, mg/dL, median (range) | NA | 3.63 (0.44-7.08) | 215 | 3.67 (0.44-7.08) | 174 |
| Creatinine, mg/dL, median (range) | NA | 1.06 (0.45-11.96) | 273 | 1.06 (0.45-10.5) | 229 |
| B2M, mg/L, median (range) | NA | 3.8 (0.61-44.32) | 247 | 3.8 (0.61-44.32) | 207 |
| Hemoglobin, g/dL, median (range) | NA | 11.1 (4.9-17.5) | 247 | 11.1 (4.9-17.5) | 203 |
| Platelets, G/L, median (range) | NA | 215 (3.93-481) | 249 | 215 (3.93-481) | 204 |
| Calcium, mmol/L, median (range) | NA | 2.29 (1.27-5) | 260 | 2.28 (1.27-5) | 219 |
| BM plasma cell^a^, %, median (range) | NA | 33.25 (0.5-100) | 754 | 40 (0.5-99) | 281 |
| SFLC involved/uninvolved ratio, median (range) | NA | 41.44 (1.03-40000) | 222 | 32.48 (1.03-40000) | 188 |
| Female gender, n (%) | NA | 373 (47) | 794 | 127 (42) | 299 |
| Samples from pretreated patients, n (%) | NA | 135 (17) | 794 | 53 (18) | 299 |
| MACS enriched samples, n (%) | NA | 344 (43) | 794 | 84 (28) | 299 |
| High-risk CA^b^, n (%) | NA | 158 (20) | 794 | 61 (20) | 299 |
| ISS, n (%) | I | 62 (27) | 226 | 51 (27) | 187 |
|  | II | 92 (41) |  | 79 (42) |  |
|  | III | 72 (32) |  | 57 (30) |  |
| R-ISS, n (%) | I | 42 (19) | 219 | 35 (19) | 180 |
|  | II | 144 (66) |  | 121 (67) |  |
|  | III | 33 (15) |  | 24 (13) |  |
| M-protein isotype, n (%) | IgG | 203 (56) | 364 | 157 (55) | 285 |
|  | IgA | 81 (23) |  | 65 (23) |  |
|  | Light chain only | 76 (21) |  | 59 (21) |  |
|  | Other | 4 (1) |  | 4 (1) |  |
| Light chain kappa, n (%) | NA | 239 (65) | 370 | 182 (63) | 287 |
| Primary PCL, n (%) | NA | 13 (4) | 328 | 10 (3) | 289 |
| Secondary PCL, n (%) | NA | 11 (3) |  | 8 (3) |  |
| Primary EMM^c^, n (%) | NA | 26 (8) |  | 24 (8) |  |
| Secondary EMM^c^, n (%) | NA | 26 (8) |  | 25 (9) |  |
| AL, n (%) | NA | 14 (4) | 323 | 12 (4) | 289 |
| Induction therapy, n (%) | IMiD-based induction | 14 (5) | 311 | 14 (5) | 291 |
|  | PI-based induction | 123 (40) |  | 116 (40) |  |
|  | PI- and IMiD-based induction | 165 (53) |  | 153 (53) |  |
|  | Other | 9 (3) |  | 8 (3) |  |
| Maintenance therapy, n (%) | NA | 111 (39) | 287 | 104 (37) | 280 |
| Frontline ASCT, n (%) | NA | 135 (44) | 307 | 129 (45) | 288 |
| *Note:* Clinical features at diagnosis, unless otherwise indicated.  Abbreviations: AL, amyloidosis; ASCT, autologous stem cell transplantation; BM, bone marrow; B2M, beta-2-microglobulin; IMiD, immunomodulatory drugs; ISS, International Staging System^1^; LDH, lactate dehydrogenase; MACS, magnetic-activated cell sorting; NA, not applicable; PCL, plasma cell leukemia; PI, proteasome inhibitor; R-ISS, revised International Staging System^2^; SFLC, serum free light chain.  ^a^Clinical feature at time of first sampling.  ^b^High-risk chromosomal abnormalities (CA) were defined as del(17p), t(4;14), and t(14;16).  ^c^Extramedullary myeloma (EMM) was defined as plasma cell infiltration of the soft tissue (extramedullary extraosseous and/or extramedullary bone-related). | | | | | |

| **Table S2. Associations between subgroups and cytogenetic aberrations and immunophenotypic findings** | | | | | | | | | | | | |
| --- | --- | --- | --- | --- | --- | --- | --- | --- | --- | --- | --- | --- |
|  | All cases  (n=794) | | CG11q^a^ cases  (n=271) | | t(4;14) cases  (n=63) | | t(11;14) cases  (n=146) | | t(14;16) cases  (n=19) | | Remaining cases  (n=295) | |
| Variable | n/N | | n/N | | n/N | | n/N | | n/N | | n/N | |
| *Common CA* | | | | | | | | | | | | |
| del(1p36) | 11/432 | 3% | 0/147 | 0% | 0/34 | 0% | 0/58 | 0% | 0/8 | 0% | **11/185** | **6% **** |
| del(1p32) | 32/362 | 9% | 9/124 | 7% | 4/29 | 14% | 2/88 | 2% | 2/11 | 18% | 15/110 | 14% |
| gain(1p32) *(≥3 copies)* | 20/362 | 6% | 7/124 | 6% | 3/29 | 10% | 3/88 | 3% | 3/11 | 27% | 4/110 | 4% |
| gain(1p36) *(≥3 copies)* | 22/432 | 5% | 4/147 | 3% | 2/34 | 6% | 4/58 | 7% | **3/8** | **38% *** | 9/185 | 5% |
| gain(1q) *(≥3 copies)* | 336/794 | 42% | **78/271** | **29% **** | **45/63** | **71% **** | **36/146** | **25% **** | 12/19 | 63% | **165/295** | **56% **** |
| del(11q) | 14/794 | 2% | **0/271** | **0% *** | 4/63 | 6% | 0/146 | 0% | 0/19 | 0% | 10/295 | 3% |
| gain(11q) *(≥3 copies)* | 375/794 | 47% | **271/271** | **100% **** | **12/63** | **19% **** | **49/146** | **34% *** | 8/19 | 42% | **35/295** | **12% **** |
| del(13q) | 356/794 | 45% | **64/271** | **24% **** | **49/63** | **78% **** | **48/146** | **33% *** | 13/19 | 68% | **182/295** | **62% **** |
| gain(13q) *(≥3 copies)* | 15/794 | 2% | 7/271 | 3% | 2/63 | 3% | 1/146 | 1% | 2/19 | 11% | 3/295 | 1% |
| del(14q) | 42/794 | 5% | 9/271 | 3% | 0/63 | 0% | **0/146** | **0% *** | 0/19 | 0% | **33/295** | **11% **** |
| Unspecified IGH abnormality | 223/794 | 28% | 81/271 | 30% | **0/63** | **0% **** | **0/146** | **0% **** | **0/19** | **0% *** | **142/295** | **48% **** |
| del(17p) | 82/794 | 10% | 24/271 | 9% | 4/63 | 6% | 12/146 | 8% | 2/19 | 11% | 40/295 | 14% |
| gain(17p) *(≥3 copies)* | 80/794 | 10% | **41/271** | **15% *** | 6/63 | 10% | 7/146 | 5% | 6/19 | 32% | 20/295 | 7% |
| *Amplifications* | | | | | | | | | | | | |
| gain(1p36) *(≥4 copies)* | 9/432 | 2% | 1/147 | 1% | 1/34 | 3% | 2/58 | 3% | 2/8 | 25% | 3/185 | 2% |
| gain(1p32) *(≥4 copies)* | 7/362 | 2% | 1/124 | 1% | 1/29 | 3% | 2/88 | 2% | 1/11 | 9% | 2/110 | 2% |
| gain(1q) *(≥4 copies)* | 119/794 | 15% | **21/271** | **8% **** | **19/63** | **30% *** | **11/146** | **8% *** | **10/19** | **53% *** | **58/295** | **20% *** |
| gain(1q) *(≥5 copies)* | 30/794 | 4% | 7/271 | 3% | 3/63 | 5% | 1/146 | 1% | 3/19 | 16% | 16/295 | 5% |
| gain(11q) *(≥4 copies)* | 89/794 | 11% | **60/271** | **22% **** | 6/63 | 10% | 11/146 | 8% | 6/19 | 32% | **6/295** | **2% **** |
| gain(17p) *(≥4 copies)* | 21/794 | 3% | 4/271 | 1% | 4/63 | 6% | 3/146 | 2% | **6/19** | **32% **** | 4/295 | 1% |
| Tetraploidy^b^ | 23/794 | 3% | 5/271 | 2% | 4/63 | 6% | 4/146 | 3% | **6/19** | **32% **** | 4/295 | 1% |
| Hyperdiploidy^c^ | 65/119 | 55% | **40/45** | **89% **** | 2/10 | 20% | **4/25** | **16% **** | 3/4 | 75% | 16/35 | 46% |
| *High-risk groups* | | | | | | | | | | | | |
| High-risk CA^d^ | 158/794 | 20% | **24/271** | **9% **** | **63/63** | **100% **** | **12/146** | **8% **** | **19/19** | **100% **** | **40/295** | **14% *** |
| R-ISS III | 33/219 | 15% | 5/69 | 7% | **9/25** | **36% *** | 7/43 | 16% | 1/4 | 25% | 11/78 | 14% |
| Double-hit^e^ | 87/794 | 11% | **6/271** | **2% **** | **46/63** | **73% **** | **5/146** | **3% *** | **13/19** | **68% **** | **17/295** | **6% *** |
| Triple-hit^e^ | 4/794 | 1% | 0/271 | 0% | **3/63** | **5% *** | 0/146 | 0% | 1/19 | 5% | 0/295 | 0% |
| gain(1q) (≥4 copies) plus ISS III^f^ | 20/235 | 9% | 7/76 | 9% | 4/26 | 15% | 3/45 | 7% | 0/5 | 0% | 6/83 | 7% |
| *Immunophenotype*^g^ | | | | | | | | | | | | |
| CD38 | 317/318 | 100% | 106/107 | 99% | 28/28 | 100% | 60/60 | 100% | 7/7 | 100% | 116/116 | 100% |
| CD138 | 269/278 | 97% | 90/92 | 98% | 26/26 | 100% | 46/50 | 92% | 6/6 | 100% | 101/104 | 97% |
| CD45 | 36/235 | 15% | 15/74 | 20% | 0/24 | 0% | 6/43 | 14% | 3/7 | 43% | 12/87 | 14% |
| CD19 | 6/312 | 2% | 2/104 | 2% | 0/28 | 0% | 1/58 | 2% | 0/7 | 0% | 3/115 | 3% |
| CD117 | 108/255 | 42% | **57/87** | **66% **** | **2/24** | **8% *** | 14/49 | 29% | 3/5 | 60% | 32/90 | 36% |
| CD56 | 190/280 | 68% | **86/97** | **89% **** | 23/27 | 85% | **18/50** | **36% **** | **0/6** | **0% *** | 63/100 | 63% |
| CD27 | 88/149 | 59% | 28/46 | 61% | 7/13 | 54% | 19/35 | 54% | 1/4 | 25% | 33/51 | 65% |
| CD28 | 41/135 | 30% | 10/37 | 27% | 0/8 | 0% | 14/34 | 41% | 2/2 | 100% | 15/54 | 28% |
| CD81 | 47/147 | 32% | 13/45 | 29% | 3/12 | 25% | 12/34 | 35% | 2/3 | 67% | 17/53 | 32% |
| Co-expression CD56/CD117 | 72/303 | 24% | **50/100** | **50% **** | **1/28** | **4% *** | **1/61** | **2% **** | 0/6 | 0% | 20/108 | 19% |
| Abbreviations: CA, chromosomal abnormalities; ISS, International Staging System^1^; R-ISS, revised International Staging System^2^.  ^a^Subgroup clonal gain(11q) (CG11q) was defined as the presence of clonal gain(11q) and the absence of t(4;14), t(11;14), and t(14;16).  ^b^Tetraploidy was defined as three or more amplified regions (four or more copies) with the standard FISH panel [1p36 (D1S2795,D1S253) or 1p32 (CDKN2C), 1q21 (CKS1B), 11q22 (ATM), 13q14 (DLEU1), 14q32 (IGH), and 17p13 (TP53)].  ^c^Hyperdiploidy was defined as a gain of any two of the chromosomal regions 5p15, 9q22, and 15q22.^3^  ^d^High-risk CA were defined as del(17p), t(4;14), and t(14;16).^2^  ^e^Double-hit and triple-hit myeloma were defined as the co-occurrence of two or three adverse lesions, respectively.^4,5^ The translocations t(4;14), t(14;16),gain(1q) and del(17p) were counted as adverse; t(14;20) was not tested.  ^f^High-risk feature defined by Walker *et al.*^6^  ^g^Immunophenotyping: only homogeneous antigen expression was considered in the analysis.  **P* < 0.05; ***P* < 0.001, two-sided Fisher’s exact test. For multiple testing *P* values were adjusted with the Benjamini-Hochberg method. | | | | | | | | | | | | |

| **Table S3. Associations between subgroups and clinical findings** | | | | | | | | | | | | |
| --- | --- | --- | --- | --- | --- | --- | --- | --- | --- | --- | --- | --- |
|  | All cases (N=794) | | CG11q^a^ cases (N=271) | | t(4;14) cases (N=63) | | t(11;14) cases (N=146) | | t(14;16) cases (N=19) | | Remaining cases (N=295) | |
| Variable | n/N | | n/N | | n/N | | n/N | | n/N | | n/N | |
| Female gender | 373/794 | 47% | **106/271** | **39% *** | 33/63 | 52% | 65/146 | 45% | **15/19** | **79% *** | 154/295 | 52% |
| Age (≥ 65 years)^b^ | 536/794 | 68% | 187/271 | 69% | 39/63 | 62% | 93/146 | 64% | 14/19 | 74% | 203/295 | 69% |
| Bone marrow plasma cell (>60%)^b^ | 175/754 | 23% | 50/253 | 20% | 13/63 | 21% | 45/142 | 32% | 8/19 | 42% | 59/277 | 21% |
| Samples from pretreated patients | 135/794 | 17% | 48/271 | 18% | 9/63 | 14% | 25/146 | 17% | 3/19 | 16% | 50/295 | 17% |
| IgG | 203/365 | 56% | **84/127** | **66% *** | 20/32 | 62% | **24/66** | **36% *** | 3/8 | 38% | 72/132 | 55% |
| IgA | 81/365 | 22% | 28/127 | 22% | 10/32 | 31% | 11/66 | 17% | 3/8 | 38% | 29/132 | 22% |
| Light chain only | 76/365 | 21% | **15/127** | **12% *** | 2/32 | 6% | **30/66** | **45% **** | 2/8 | 25% | 27/132 | 20% |
| Other^c^ | 4/365 | 1% | 0/127 | 0% | 0/32 | 0% | 1/66 | 2% | 0/8 | 0% | 3/132 | 2% |
| Kappa | 239/370 | 65% | 89/125 | 71% | 16/32 | 50% | 41/69 | 59% | 7/9 | 78% | 86/135 | 64% |
| Lambda | 127/370 | 34% | 36/125 | 29% | 16/32 | 50% | 27/69 | 39% | 2/9 | 22% | 46/135 | 34% |
| LDH (increased) | 61/264 | 23% | 15/87 | 17% | 5/27 | 19% | 13/51 | 25% | 2/6 | 33% | 26/93 | 28% |
| Creatinine (≥ 2 mg/dL) | 46/273 | 17% | 15/92 | 16% | 3/26 | 12% | 9/53 | 17% | 0/7 | 0% | 19/95 | 20% |
| B2M (≥ 5.5 mg/L) | 74/247 | 30% | 19/80 | 24% | 10/26 | 38% | 16/46 | 35% | 1/5 | 20% | 28/90 | 31% |
| Hemoglobin (< 10 g/dL) | 85/247 | 34% | 25/79 | 32% | 7/26 | 27% | 16/52 | 31% | 6/7 | 86% | 31/83 | 37% |
| Platelets (< 150 G/L) | 57/249 | 23% | 17/79 | 22% | 5/26 | 19% | 10/52 | 19% | 4/7 | 57% | 21/85 | 25% |
| Calcium (≥ 2.75 mmol/L) | 17/260 | 7% | 5/88 | 6% | 1/25 | 4% | 2/48 | 4% | 0/7 | 0% | 9/92 | 10% |
| Involved/uninvolved SFLC ratio (≥100) | 91/222 | 41% | 31/76 | 41% | 12/23 | 52% | 20/42 | 48% | 0/3 | 0% | 28/78 | 36% |
| ISS I | 66/236 | 28% | 26/77 | 34% | 8/26 | 31% | 14/45 | 31% | 1/5 | 20% | 17/83 | 20% |
| ISS II | 96/236 | 41% | 31/77 | 40% | 9/26 | 35% | 16/45 | 36% | 3/5 | 60% | 37/83 | 45% |
| ISS III | 74/236 | 31% | 20/77 | 26% | 9/26 | 35% | 15/45 | 33% | 1/5 | 20% | 29/83 | 35% |
| PI-based induction | 123/311 | 40% | 45/107 | 42% | 11/28 | 39% | 20/61 | 33% | 2/7 | 29% | 45/108 | 42% |
| IMiD-based induction | 14/311 | 5% | 7/107 | 7% | 0/28 | 0% | 1/61 | 2% | 0/7 | 0% | 6/108 | 6% |
| PI- and IMiD-based induction | 165/311 | 53% | 52/107 | 49% | 17/28 | 61% | 37/61 | 61% | 5/7 | 71% | 54/108 | 50% |
| Other^d^ | 9/311 | 3% | 3/107 | 3% | 0/28 | 0% | 3/61 | 5% | 0/7 | 0% | 3/108 | 3% |
| Maintenance/Consolidation | 82/181 | 45% | 33/72 | 46% | 9/13 | 69% | 15/29 | 52% | 1/4 | 25% | 24/63 | 38% |
| Front-line ASCT | 119/233 | 51% | 46/87 | 53% | 12/17 | 71% | 22/42 | 52% | 1/5 | 20% | 38/82 | 46% |
| EMM^e^ | 52/328 | 16% | 15/111 | 14% | 7/33 | 21% | 8/63 | 13% | 2/9 | 22% | 20/112 | 18% |
| PCL^f^ | 24/328 | 7% | 3/111 | 3% | 5/33 | 15% | 7/63 | 11% | **4/9** | **44% *** | 5/112 | 4% |
| AL | 14/323 | 4% | 4/110 | 4% | 2/32 | 6% | 4/62 | 6% | 0/7 | 0% | 4/112 | 4% |
| *Note:* Clinical features at diagnosis, unless otherwise indicated.  Abbreviations: AL, amyloidosis; ASCT, autologous stem cell transplantation; B2M, beta-2 microglobulin; IMiD, immunomodulatory drugs; ISS, International Staging System^1^; LDH, lactate dehydrogenase; PI, proteasome inhibitor; SFLC, serum free light chain. ^a^Subgroup clonal gain(11q) (CG11q) was defined as the presence of clonal gain(11q) and the absence of t(4;14), t(11;14), and t(14;16). ^b^Clinical feature at time of first sampling.  ^c^This category includes IgD, IgM, and non-secretory MM. ^d^This category includes alkylating agents and monoclonal antibodies. ^e^Extramedullary myeloma (EMM) was present at diagnosis or developed during disease course and was defined as plasma cell infiltration of the soft tissue (extramedullary extraosseous and/or extramedullary bone-related). ^f^Plasma cell leukemia (PCL) was present at diagnosis or developed during disease course. **P* < 0.05; ***P* < 0.001, Fisher’s exact test. For multiple testing *P* values were adjusted with the Benjamini-Hochberg method. | | | | | | | | | | | | |

| **Table S4. Pairwise associations** | | | | |
| --- | --- | --- | --- | --- |
| Variable 1 | Variable 2 | Odds ratio | P value | Corrected P value (BH) |
| amp1q | del1p32 | 6.44948494 | 3.7979E-06 | 5.2963E-05 |
| gain_amp1q | del1p32 | 4.57122268 | 9.8018E-05 | 0.00111087 |
| del17p | del1p32 | 5.72853822 | 0.00078039 | 0.00740959 |
| del14q | del1p36 | 25.3702695 | 0.00014634 | 0.00159284 |
| del13q | del1p36 | 12.3390918 | 0.00341514 | 0.02685472 |
| Female gender | del13q | 2.08967376 | 3.6521E-07 | 5.7946E-06 |
| Involved/uninvolved SFLC ratio (≥100) | Female gender | 2.42651501 | 0.00155893 | 0.01364061 |
| Light chain only myeloma | Female gender | 2.16022615 | 0.00409917 | 0.03126928 |
| CG11q without (1q) | CD56_CD117comb | 5.22512934 | 5.2212E-08 | 8.834E-07 |
| CG11q plus gain(1q) | CD56_CD117comb | 3.63806611 | 0.00060066 | 0.00589113 |
| B2M (≥ 5.5 mg/L) | Creatinine (≥ 2 mg/dL) | 141.4602431 | 9.95361E-20 | 3.01139E-18 |
| Hemoglobin (< 10 g/dL) | B2M (≥ 5.5 mg/L) | 4.690416079 | 1.17075E-06 | 1.76354E-05 |
| SFLC ratio (≥100) | B2M (≥ 5.5 mg/L) | 5.42320404 | 1.17823E-06 | 1.76734E-05 |
| Platelets (< 150 G/L) | B2M (≥ 5.5 mg/L) | 4.46225461 | 2.26349E-05 | 0.000278644 |
| Calcium (≥ 2.75 mmol/L) | B2M (≥ 5.5 mg/L) | 7.256191253 | 0.000486365 | 0.00487731 |
| This table lists the statistically additional significant findings cited in the manuscript. In total 415 of the 3487 pairs were statistically significant following Fisher’s exact test and Benjamini-Hochberg (BH) correction (*P* < .05). | | | | |

| **Table S5. Association between cytogenetic, immunophenotypic, and clinical features and TTNT/OS.** | | | | |
| --- | --- | --- | --- | --- |
|  | TTNT | | OS | |
| Variable | HR (95% CI) | *P* value | HR (95% CI) | *P* value |
| del(1p36) | 2.5 (0.92-6.8) | 0.072 | 1.7 (0.53-5.3) | 0.38 |
| gain(1p36) (3 copies)^a^ | 1.2 (0.49-2.9) | 0.7 | 1.3 (0.46-3.4) | 0.65 |
| del(1p32) | 1.8 (0.74-4.2) | 0.2 | 3.5 (1.3-9.1) | **0.011** |
| gain(1p32) (3 copies)^a^ | 1.2 (0.28-5.1) | 0.81 | 2.8 (0.64-12) | 0.17 |
| gain(1q) (3 copies)^a^ | 1.5 (1.1-2) | **0.0095** | 1.7 (1.1-2.4) | **0.0086** |
| del(11q) | 0.98 (0.36-2.6) | 0.97 | 1.1 (0.28-4.6) | 0.86 |
| gain(11q) (3 copies)^a^ | 1.1 (0.79-1.4) | 0.71 | 0.99 (0.69-1.4) | 0.96 |
| del(13q) | 1.5 (1.1-2) | **0.0035** | 1.7 (1.2-2.5) | **0.0013** |
| del(14q) | 0.61 (0.22-1.6) | 0.32 | 0.95 (0.35-2.6) | 0.93 |
| Unspecified IGH alteration | 0.82 (0.6-1.1) | 0.22 | 1.1 (0.72-1.5) | 0.78 |
| del(17p) | 1.7 (1.1-2.6) | **0.0088** | 2.3 (1.4-3.7) | **0.00044** |
| gain(17p) (3 copies)^a^ | 0.6 (0.33-1.1) | 0.11 | 0.79 (0.39-1.6) | 0.53 |
| t(4;14) subgroup | 1.7 (1.1-2.7) | **0.018** | 1.8 (0.98-3.1) | 0.058 |
| t(11;14) subgroup | 1.2 (0.84-1.6) | 0.35 | 1.3 (0.85-1.9) | 0.23 |
| t(14;16) subgroup | 2.5 (1.1-5.7) | **0.026** | 2 (0.83-5) | 0.12 |
| CG11q^b^ subgroup | 0.75 (0.57-1) | 0.053 | 0.66 (0.46-0.95) | **0.025** |
| Remaining cases subgroup | 0.93 (0.7-1.2) | 0.65 | 0.99 (0.7-1.4) | 0.97 |
| gain(1p36) (≥ 4 copies)^a^ | 0.99 (0.6-1.6) | 0.97 | 1.2 (0.69-2.2) | 0.49 |
| gain(1p32) (≥ 4 copies)^a^ | 6.4 (1.9-21) | **0.0024** | 9.1 (2.3-36) | **0.0018** |
| gain(1q) (≥ 4 copies)^a^ | 1.5 (1.2-1.8) | **6.9e-05** | 1.6 (1.3-2) | **7.4e-05** |
| gain(11q) (≥ 4 copies)^a^ | 1.1 (0.85-1.4) | 0.52 | 1 (0.76-1.3) | 0.95 |
| gain(17p) (≥ 4 copies)^a^ | 1.1 (0.68-1.7) | 0.79 | 1.2 (0.72-1.9) | 0.52 |
| Early tetraploidy^c^ | 1.6 (0.75-3.4) | 0.23 | 2 (0.88-4.6) | 0.097 |
| Hyperdiploidy^d^ | 0.74 (0.13-4.1) | 0.73 | 1.6 (0.21-12) | 0.66 |
| High-risk CA^e^ | 1.9 (1.4-2.6) | **9.8e-05** | 2.2 (1.5-3.2) | **6.1e-05** |
| Double-hit^f^ | 2.3 (1.5-3.4) | **5.6e-05** | 2.8 (1.8-4.5) | **1.2e-05** |
| Triple-hit^f^ | 10 (2.4-42) | **0.0014** | 9 (2.2-37) | **0.0025** |
| gain(1q) (≥ 4 copies) + ISS III^g^ | 3.3 (1.9-5.7) | **2.4e-05** | 4.7 (2.5-8.8) | **2.1e-06** |
| Female gender | 1.4 (1-1.8) | **0.026** | 1.2 (0.88-1.7) | 0.21 |
| Age (≥ 65 years)^h^ | 1.3 (1-1.7) | 0.051 | 1.8 (1.2-2.5) | **0.0019** |
| BM plasma cell (>60%)^h^ | 1.3 (0.98-1.8) | 0.07 | 1.6 (1.1-2.4) | **0.01** |
| IgG | 0.81 (0.61-1.1) | 0.13 | 0.84 (0.59-1.2) | 0.31 |
| IgA | 1.4 (0.98-1.9) | 0.068 | 1.5 (1-2.2) | **0.048** |
| Light chain only | 0.96 (0.69-1.3) | 0.82 | 0.81 (0.52-1.2) | 0.33 |
| Other^i^ | 2.1 (0.79-5.8) | 0.13 | 2 (0.65-6.4) | 0.22 |
| Kappa | 1.1 (0.82-1.5) | 0.51 | 0.85 (0.59-1.2) | 0.39 |
| Lambda | 0.88 (0.66-1.2) | 0.41 | 1.1 (0.77-1.6) | 0.57 |
| LDH (increased) | 1.2 (0.81-1.7) | 0.39 | 1.5 (0.97-2.4) | 0.067 |
| Creatinine (≥ 2 mg/dL) | 2 (1.3-2.9) | **0.00075** | 2.5 (1.6-4) | **0.00011** |
| B2M (≥ 5.5 mg/L) | 2.1 (1.5-3) | **4.6e-05** | 3.2 (2-4.9) | **3.3e-07** |
| Hemoglobin (< 10 g/dL) | 1.6 (1.1-2.2) | **0.011** | 2.1 (1.4-3.1) | **0.00038** |
| Platelets (< 150 G/L) | 1.6 (1.1-2.4) | **0.011** | 2.5 (1.6-3.9) | **7.4e-05** |
| Calcium (≥ 2.75 mmol/L) | 2 (1.1-3.6) | **0.025** | 2.8 (1.4-5.4) | **0.0023** |
| Involved/uninvolved SFLC ratio (≥100) | 1.5 (1-2.1) | **0.034** | 2 (1.3-3.1) | **0.0016** |
| ISS I | 0.59 (0.41-0.86) | **0.006** | 0.43 (0.26-0.72) | **0.0015** |
| ISS II | 0.91 (0.65-1.3) | 0.6 | 0.86 (0.57-1.3) | 0.48 |
| ISS III | 2 (1.4-2.9) | **8.7e-05** | 2.9 (1.9-4.5) | **2.1e-06** |
| R-ISS I | 0.47 (0.29-0.74) | **0.0013** | 0.34 (0.17-0.67) | **0.0021** |
| R-ISS II | 1.2 (0.85-1.8) | 0.28 | 1 (0.64-1.6) | 0.93 |
| R-ISS III | 2.5 (1.5-4.1) | **0.00021** | 5.1 (2.9-9) | **3.1e-08** |
| PI-based induction | 1.5 (1.2-2) | **0.0027** | 1.7 (1.2-2.3) | **0.004** |
| IMiD-based induction | 0.81 (0.42-1.6) | 0.54 | 0.83 (0.36-1.9) | 0.65 |
| PI- and IMiD-based induction | 0.79 (0.6-1) | 0.093 | 0.71 (0.5-1) | 0.057 |
| Other^j^ | 0.47 (0.22-1) | 0.054 | 0.5 (0.2-1.3) | 0.15 |
| Front-line ASCT^k^ | 0.58 (0.42-0.8) | **0.00088** | 0.52 (0.34-0.79) | **0.0023** |
| Maintenance therapy^k^ | 0.83 (0.56-1.2) | 0.33 | 1.3 (0.75-2.2) | 0.35 |
| AL | 1.2 (0.61-2.5) | 0.55 | 3.3 (1.6-6.8) | **0.0012** |
| CD38 | 0.26 (0.081-0.81) | **0.021** | 0.52 (0.13-2.1) | 0.36 |
| CD138 | 0.99 (0.64-1.5) | 0.96 | 1.6 (0.89-2.8) | 0.12 |
| CD45 | 1.1 (0.71-1.8) | 0.58 | 0.94 (0.51-1.7) | 0.84 |
| CD19 | 1.5 (0.49-4.9) | 0.46 | 0.53 (0.074-3.8) | 0.53 |
| CD117 | 0.89 (0.62-1.3) | 0.55 | 0.9 (0.56-1.4) | 0.66 |
| CD56 | 0.92 (0.67-1.3) | 0.63 | 0.77 (0.52-1.1) | 0.19 |
| CD27 | 0.57 (0.34-0.95) | **0.031** | 0.44 (0.2-0.96) | **0.04** |
| CD28 | 1.9 (1-3.3) | **0.034** | 1.5 (0.67-3.5) | 0.31 |
| CD81 | 2.2 (1.3-3.8) | **0.0039** | 2 (1-4.1) | 0.051 |
| *Note:* Clinical features at diagnosis, unless otherwise indicated.  Abbreviations: AL, amyloidosis; ASCT, autologous stem cell transplantation; BM, bone marrow; B2M, beta-2 microglobulin; CI, confidence interval; HR, hazard ratio; IMiD, immunomodulatory drugs; ISS, International Staging System^1^; LDH, lactate dehydrogenase;  OS, overall survival; PI, proteasome inhibitors; R-ISS, revised International Staging System^2^; SFLC, serum free light chain; TTNT, time to next treatment. ^a^Versus no gain (≤2 copies).  ^b^Subgroup clonal gain(11q) (CG11q) was defined as the presence of clonal gain(11q) and the absence of t(4;14), t(11;14), and t(14;16).  ^c^Tetraploidy was defined as 3 or more amplified regions (4 or more copies) with the standard FISH panel [1p36 (D1S2795,D1S253) or 1p32 (CDKN2C), 1q21 (CKS1B), 11q22 (ATM), 13q14 (DLEU1), 14q32 (IGH), and 17p13 (TP53)]. ^d^Hyperdiploidy was defined as a gain of any two of the chromosomal regions 5p15, 9q22, and 15q22.^3^  ^e^High-risk chromosomal abnormalities (CA) were defined as del(17p), t(4;14), and t(14;16).^2^  ^f^Double-hit and triple-hit myeloma were defined as the co-occurrence of two or three adverse lesions, respectively.^4,5^ The translocations t(4;14), t(14;16),gain(1q) and del(17p) were counted as adverse; t(14;20) was not tested.  ^g^High-risk feature defined by Walker *et al.*^6^  ^h^Clinical feature at the time of the first sampling. ^i^This category includes IgD, IgM, and non-secretory MM. ^j^This category includes alkylating agents and monoclonal antibodies. ^k^Only cases with a TTNT/OS >= 6 months (ASCT) or >= 12 months (maintenance) were considered. Hazard ratios (HR) and *P* values were derived using univariate Cox regression analysis. | | | | |

| **Table S6. Association between cytogenetic, immunophenotypic, and clinical features and TTNT/OS in the CG11q^a^ subgroup.** | | | | |
| --- | --- | --- | --- | --- |
|  | TTNT | | OS | |
|  |  |  |  |  |
| Variable | HR (95% CI) | *P* value | HR (95% CI) | *P* value |
| del(1p36) | NA (NA-NA) |  | NA (NA-NA) |  |
| gain(1p36) (3 copies)^b^ | 3.8e-08 (0-Inf) | 1 | 1.1e-07 (0-Inf) | 1 |
| del(1p32) | 3.2 (0.68-15) | 0.14 | 4.2 (0.73-25) | 0.11 |
| gain(1p32) (3 copies)^b^ | 0.65 (0.073-5.8) | 0.7 | 0.91 (0.082-9.9) | 0.93 |
| gain(1q) (3 copies)^b^ | 2.6 (1.5-4.4) | **0.00052** | 4 (2.1-7.9) | **4.3e-05** |
| del(11q) | NA (NA-NA) |  | NA (NA-NA) |  |
| gain(11q) (3 copies)^b^ | NA (NA-NA) |  | NA (NA-NA) |  |
| del(13q) | 1.2 (0.67-2.1) | 0.58 | 1.6 (0.77-3.3) | 0.21 |
| del(14q) | 2.4 (0.33-18) | 0.39 | 1.6 (0.22-12) | 0.65 |
| Unspecified IGH alteration | 1 (0.58-1.7) | 0.99 | 1.3 (0.62-2.7) | 0.5 |
| del(17p) | 1.6 (0.8-3.3) | 0.18 | 1.6 (0.61-4) | 0.36 |
| gain(17p) (3 copies)^b^ | 0.78 (0.37-1.6) | 0.52 | 1 (0.39-2.6) | 1 |
| gain(1p36) (≥ 4 copies)^b^ | 5.8 (1.8-19) | **0.004** | 3.3 (1.1-9.5) | **0.028** |
| gain(1p32) (≥ 4 copies)^b^ | 0.65 (0.073-5.8) | 0.7 | 0.91 (0.082-9.9) | 0.93 |
| gain(1q) (≥ 4 copies)^b^ | 1.6 (1-2.5) | **0.036** | 2.3 (1.4-3.9) | **0.002** |
| gain(11q) (≥ 4 copies)^b^ | NA (NA-NA) |  | NA (NA-NA) |  |
| gain(17p) (≥ 4 copies)^b^ | 1.3 (0.47-3.4) | 0.64 | 1.7 (0.6-4.6) | 0.32 |
| Early tetraploidy^c^ | 1.7 (0.23-12) | 0.6 | 3 (0.4-23) | 0.28 |
| Hyperdiploidy^d^ | 0.41 (0.025-6.6) | 0.53 | 0.41 (0.025-6.6) | 0.53 |
| High-risk CA^e^ | 1.6 (0.8-3.3) | 0.18 | 1.6 (0.61-4) | 0.36 |
| Double-hit^f^ | 4.2 (1.3-14) | **0.016** | 5.1 (1.5-17) | **0.0074** |
| Triple-hit^f^ | NA (NA-NA) |  | NA (NA-NA) |  |
| gain(1q) (≥ 4 copies) + ISS III^g^ | 3 (1-8.6) | **0.045** | 4.2 (1.2-15) | **0.029** |
| Female gender | 1.3 (0.79-2.1) | 0.31 | 1 (0.55-2) | 0.88 |
| Age (≥ 65 years)^h^ | 2 (1.2-3.2) | **0.0042** | 3.5 (1.7-7.2) | **0.00062** |
| BM plasma cell (>60%)^h^ | 1.4 (0.78-2.4) | 0.27 | 2.7 (1.4-5.4) | **0.0034** |
| IgG | 0.66 (0.4-1.1) | 0.11 | 0.9 (0.46-1.8) | 0.76 |
| IgA | 1.6 (0.88-2.8) | 0.13 | 1.5 (0.7-3) | 0.32 |
| Light chain only | 1.2 (0.59-2.4) | 0.61 | 0.68 (0.24-1.9) | 0.47 |
| Other^i^ | NA (NA-NA) |  | NA (NA-NA) |  |
| Kappa | 1.1 (0.67-2) | 0.63 | 0.78 (0.38-1.6) | 0.48 |
| Lambda | 0.88 (0.51-1.5) | 0.63 | 1.3 (0.64-2.6) | 0.48 |
| LDH (increased) | 0.8 (0.32-2) | 0.64 | 0.68 (0.16-2.9) | 0.6 |
| Creatinine (≥ 2 mg/dl) | 4.2 (2-8.9) | **0.00017** | 4.5 (1.7-12) | **0.0027** |
| B2M (≥ 5.5 mg/l) | 4.1 (1.9-8.5) | **0.00018** | 4.6 (1.6-13) | **0.0038** |
| Hemoglobin (< 10 g/dl) | 1.7 (0.88-3.2) | 0.11 | 2.2 (0.97-4.8) | 0.06 |
| Platelets (< 150 G/l) | 1.9 (0.9-4) | 0.091 | 1.9 (0.69-5.3) | 0.22 |
| Calcium (≥ 2.75 mmol/l) | 3.2 (1.1-9.1) | **0.028** | 2.6 (0.76-8.6) | 0.13 |
| Involved/uninvolved SFLC ratio (≥100) | 1.3 (0.69-2.5) | 0.41 | 1.8 (0.74-4.5) | 0.19 |
| ISS I | 0.4 (0.21-0.79) | **0.0077** | 0.41 (0.15-1.1) | 0.073 |
| ISS II | 1.1 (0.59-1.9) | 0.83 | 0.9 (0.4-2) | 0.8 |
| ISS III | 3.8 (1.9-7.7) | **0.00024** | 4.6 (1.7-12) | **0.0026** |
| R-ISS I | 0.4 (0.18-0.93) | **0.032** | 0.74 (0.24-2.3) | 0.6 |
| R-ISS II | 2 (0.94-4.2) | 0.071 | 1.1 (0.4-3.2) | 0.82 |
| R-ISS III | 2.1 (0.48-8.9) | 0.33 | 2.6 (0.32-20) | 0.38 |
| PI-based induction | 1.8 (1.1-2.9) | **0.017** | 2 (1.1-3.9) | **0.03** |
| IMiD-based induction | 0.91 (0.36-2.3) | 0.84 | 1.4 (0.5-4) | 0.51 |
| PI- and IMiD-based induction | 0.73 (0.45-1.2) | 0.2 | 0.52 (0.26-1) | 0.066 |
| Other^j^ | 0.4 (0.12-1.3) | 0.13 | 0.28 (0.036-2.1) | 0.22 |
| Front-line ASCT^k^ | 0.45 (0.27-0.75) | **0.0023** | 0.33 (0.15-0.71) | **0.0048** |
| Maintenance therapy^k^ | 0.91 (0.49-1.7) | 0.75 | 1.9 (0.79-4.5) | 0.15 |
| AL | 1.9 (0.59-6.1) | 0.29 | 5.3 (1.6-18) | **0.007** |
| CD38 | 0.39 (0.053-2.9) | 0.36 | 0.33 (0.044-2.5) | 0.28 |
| CD138 | 1 (0.41-2.6) | 0.94 | 1.3 (0.37-4.2) | 0.71 |
| CD45 | 1.4 (0.7-3) | 0.32 | 1.8 (0.66-4.8) | 0.25 |
| CD19 | 1.4 (0.19-10) | 0.74 | 1.1e-07 (0-Inf) | 1 |
| CD117 | 0.98 (0.55-1.7) | 0.93 | 0.72 (0.31-1.7) | 0.44 |
| CD56 | 1.3 (0.58-2.9) | 0.53 | 1 (0.39-2.7) | 0.96 |
| CD27 | 0.94 (0.37-2.4) | 0.9 | 0.83 (0.21-3.3) | 0.79 |
| CD28 | 0.94 (0.32-2.7) | 0.91 | 1.3 (0.21-7.9) | 0.77 |
| CD81 | 2.3 (0.82-6.4) | 0.11 | 7.1 (2-25) | **0.0021** |
| *Note:* Clinical features at diagnosis, unless otherwise indicated.  Abbreviations: AL, amyloidosis; ASCT, autologous stem cell transplantation; BM, bone marrow; B2M, beta-2 microglobulin; CI, confidence interval; HR, hazard ratio; IMiD, immunomodulatory drugs; ISS, International Staging System^1^; LDH, lactate dehydrogenase;  NA, not applicable; OS, overall survival; PI, proteasome inhibitors; R-ISS, revised International Staging System^2^; SFLC, serum free light chain; TTNT, time to next treatment. ^a^Subgroup clonal gain(11q) (CG11q) was defined as the presence of clonal gain(11q) and the absence of t(4;14), t(11;14), and t(14;16).  ^b^Versus no gain (2 copies or less).  ^c^Tetraploidy was defined as 3 or more amplified regions (4 or more copies) with the standard FISH panel [1p36 (D1S2795,D1S253) or 1p32 (CDKN2C), 1q21 (CKS1B), 11q22 (ATM), 13q14 (DLEU1), 14q32 (IGH), and 17p13 (TP53)].  ^d^Hyperdiploidy was defined as a gain of any two of the chromosomal regions 5p15, 9q22, and 15q22.^3^  ^e^High-risk chromosomal abnormalities (CA) were defined as del(17p), t(4;14), and t(14;16).^2^ Because t(4;14), and t(14;16) were not present in this subgroup, high-risk CA correspond to del(17p). ^f^Double-hit and triple-hit myeloma were defined as the co-occurrence of two or three adverse lesions, respectively.^4,5^ The translocations t(4;14), t(14;16),gain(1q) and del(17p) were counted as adverse; t(14;20) was not tested.  ^g^High-risk feature defined by Walker *et al.*^6^  ^h^Clinical feature at time of first sampling.  ^i^This category includes IgD, IgM, and non-secretory MM. ^j^This category includes alkylating agents and monoclonal antibodies. ^k^Only cases with a TTNT/OS >= 6 months (ASCT) or >= 12 months (maintenance) were considered. Hazard ratios (HR) and P values were derived using univariate Cox regression analysis. | | | | |

| **Table S7. Association between cytogenetic, immunophenotypic, and clinical features and TTNT/OS in the non-CG11q^a^ cases.** | | | | |
| --- | --- | --- | --- | --- |
|  | TTNT | | OS | |
| Variable | HR (95% CI) | *P* value | HR (95% CI) | *P* value |
| del(1p36) | 2.1 (0.77-5.8) | 0.14 | 1.4 (0.43-4.4) | 0.59 |
| gain(1p36) (3 copies)^b^ | 2.4 (0.96-6) | 0.061 | 1.4 (0.52-3.9) | 0.49 |
| del(1p32) | 1.5 (0.52-4.3) | 0.46 | 3.8 (1.2-12) | **0.026** |
| gain(1p32) (3 copies)^b^ | 2.7 (0.36-20) | 0.33 | 5.2 (0.66-41) | 0.12 |
| gain(1q) (3 copies)^b^ | 1.2 (0.8-1.7) | 0.43 | 0.98 (0.62-1.6) | 0.94 |
| del(11q) | 0.85 (0.31-2.3) | 0.74 | 0.97 (0.24-4) | 0.97 |
| gain(11q) (3 copies)^b^ | 2.3 (1.5-3.5) | **0.00018** | 2 (1.2-3.3) | **0.006** |
| del(13q) | 1.5 (1-2.1) | **0.029** | 1.6 (1-2.5) | **0.03** |
| del(14q) | 0.42 (0.13-1.3) | 0.14 | 0.75 (0.24-2.4) | 0.62 |
| Unspecified IGH alteration | 0.75 (0.51-1.1) | 0.16 | 0.93 (0.59-1.5) | 0.74 |
| del(17p) | 1.8 (1.1-3.1) | **0.02** | 2.8 (1.6-4.8) | **0.00023** |
| gain(17p) (3 copies)^b^ | 0.52 (0.17-1.7) | 0.27 | 0.82 (0.26-2.6) | 0.73 |
| t(4;14) | 1.5 (0.96-2.4) | 0.077 | 1.5 (0.84-2.8) | 0.17 |
| t(11;14) | 1 (0.72-1.5) | 0.85 | 1.1 (0.72-1.7) | 0.62 |
| t(14;16) | 2.3 (0.99-5.2) | 0.053 | 1.7 (0.7-4.2) | 0.24 |
| gain(1p36) (≥ 4 copies)^b^ | 0.8 (0.45-1.4) | 0.46 | 0.97 (0.48-2) | 0.93 |
| gain(1p32) (≥ 4 copies)^b^ | 7.2 (1.8-29) | **0.0051** | 96000 (0-Inf) | 1 |
| gain(1q) (≥ 4 copies)^b^ | 1.4 (1.1-1.7) | **0.0061** | 1.4 (1-1.8) | **0.024** |
| gain(11q) (≥ 4 copies)^b^ | 1.2 (0.84-1.7) | 0.31 | 1.3 (0.86-1.9) | 0.22 |
| gain(17p) (≥ 4 copies)^b^ | 0.99 (0.6-1.6) | 0.98 | 1.1 (0.61-1.9) | 0.79 |
| Early tetraploidy^c^ | 1.4 (0.63-3.3) | 0.38 | 1.8 (0.72-4.4) | 0.21 |
| Hyperdiploidy^d^ | NA (NA-NA) |  | NA (NA-NA) |  |
| High-risk CA^e^ | 1.8 (1.3-2.7) | **0.0013** | 2.2 (1.4-3.4) | **0.00044** |
| Double-hit^f^ | 1.9 (1.3-3) | **0.0024** | 2.3 (1.4-3.8) | **0.0015** |
| Triple-hit^f^ | 7.7 (1.9-32) | **0.005** | 7.5 (1.8-32) | **0.0059** |
| gain(1q) (≥ 4 copies) + ISS III^g^ | 3.4 (1.8-6.5) | **0.00022** | 5.1 (2.5-11) | **1.2e-05** |
| Female gender | 1.4 (0.99-2) | 0.056 | 1.3 (0.87-1.9) | 0.2 |
| Age (≥ 65 years)^h^ | 1 (0.73-1.4) | 0.89 | 1.3 (0.83-1.9) | 0.28 |
| BM plasma cell (>60%)^h^ | 1.3 (0.9-1.9) | 0.16 | 1.3 (0.81-2) | 0.3 |
| IgG | 0.92 (0.65-1.3) | 0.66 | 0.89 (0.59-1.3) | 0.57 |
| IgA | 1.3 (0.85-2) | 0.24 | 1.5 (0.96-2.5) | 0.076 |
| Light chain only | 0.84 (0.56-1.2) | 0.38 | 0.74 (0.46-1.2) | 0.24 |
| Other^i^ | 1.9 (0.68-5) | 0.23 | 1.8 (0.56-5.7) | 0.32 |
| Kappa | 1.1 (0.76-1.6) | 0.63 | 0.92 (0.61-1.4) | 0.71 |
| Lambda | 0.88 (0.62-1.3) | 0.5 | 1 (0.66-1.5) | 0.97 |
| LDH (increased) | 1.3 (0.81-1.9) | 0.31 | 1.7 (1-2.8) | **0.032** |
| Creatinine (≥ 2 mg/dl) | 1.5 (0.95-2.5) | 0.081 | 2 (1.2-3.5) | **0.0095** |
| B2M (≥ 5.5 mg/l) | 1.7 (1.1-2.5) | **0.016** | 2.7 (1.6-4.4) | **8.6e-05** |
| Hemoglobin (< 10 g/dl) | 1.5 (1-2.2) | 0.052 | 1.9 (1.2-3.1) | **0.0056** |
| Platelets (< 150 G/l) | 1.5 (0.96-2.4) | 0.076 | 2.4 (1.5-4) | **0.00052** |
| Calcium (≥ 2.75 mmol/l) | 1.6 (0.76-3.3) | 0.22 | 2.8 (1.3-6.3) | **0.0098** |
| Involved/uninvolved SFLC ratio (≥100) | 1.6 (1-2.4) | **0.042** | 2.2 (1.3-3.5) | **0.0023** |
| ISS I | 0.76 (0.49-1.2) | 0.23 | 0.47 (0.26-0.87) | **0.016** |
| ISS II | 0.84 (0.56-1.3) | 0.39 | 0.83 (0.51-1.4) | 0.46 |
| ISS III | 1.6 (1.1-2.5) | **0.025** | 2.4 (1.5-4) | **4e-04** |
| R-ISS I | 0.53 (0.3-0.93) | **0.027** | 0.25 (0.1-0.63) | **0.0033** |
| R-ISS II | 1 (0.65-1.5) | 1 | 0.96 (0.57-1.6) | 0.87 |
| R-ISS III | 2.4 (1.4-4.1) | **0.0011** | 4.8 (2.6-8.8) | **5.6e-07** |
| PI-based induction | 1.5 (1.1-2.1) | **0.023** | 1.6 (1-2.4) | **0.032** |
| IMiD-based induction | 0.79 (0.29-2.1) | 0.64 | 0.49 (0.12-2) | 0.32 |
| PI- and IMiD-based induction | 0.77 (0.54-1.1) | 0.13 | 0.76 (0.5-1.2) | 0.2 |
| Other^j^ | 0.55 (0.2-1.5) | 0.24 | 0.65 (0.23-1.9) | 0.42 |
| Front-line ASCT^k^ | 0.7 (0.47-1.1) | 0.091 | 0.66 (0.39-1.1) | 0.12 |
| Maintenance therapy^k^ | 0.78 (0.47-1.3) | 0.33 | 1.1 (0.52-2.2) | 0.87 |
| AL | 1 (0.42-2.5) | 0.96 | 2.5 (1-6.3) | **0.045** |
| CD38 | 0.18 (0.043-0.75) | **0.018** | 0.69 (0.095-5) | 0.71 |
| CD138 | 1 (0.64-1.7) | 0.85 | 1.8 (0.94-3.4) | 0.079 |
| CD45 | 1 (0.55-2) | 0.9 | 0.75 (0.34-1.6) | 0.46 |
| CD19 | 1.8 (0.43-7.2) | 0.43 | 0.69 (0.095-5) | 0.71 |
| CD117 | 0.98 (0.59-1.6) | 0.94 | 1.3 (0.7-2.3) | 0.43 |
| CD56 | 0.98 (0.66-1.5) | 0.93 | 0.81 (0.5-1.3) | 0.38 |
| CD27 | 0.49 (0.26-0.95) | **0.033** | 0.36 (0.13-0.98) | **0.045** |
| CD28 | 2.4 (1.1-4.8) | **0.019** | 1.6 (0.62-4.1) | 0.34 |
| CD81 | 2.2 (1.2-4.3) | **0.016** | 1.1 (0.46-2.8) | 0.79 |
| *Note:* Clinical features at diagnosis, unless otherwise indicated.  Abbreviations: AL, amyloidosis; ASCT, autologous stem cell transplantation; BM, bone marrow; B2M, beta-2 microglobulin; CI, confidence interval; HR, hazard ratio; IMiD, immunomodulatory drugs; ISS, International Staging System^1^; LDH, lactate dehydrogenase;  NA, not applicable; OS, overall survival; PI, proteasome inhibitors; R-ISS, revised International Staging System^2^; SFLC, serum free light chain; TTNT, time to next treatment. ^a^Subgroup clonal gain(11q) (CG11q) was defined as the presence of clonal gain(11q) and the absence of t(4;14), t(11;14), and t(14;16).  ^b^Versus no gain (two copies or less).  ^c^Tetraploidy was defined as three or more amplified regions (four or more copies) with the standard FISH panel [1p36 (D1S2795,D1S253) or 1p32 (CDKN2C), 1q21 (CKS1B), 11q22 (ATM), 13q14 (DLEU1), 14q32 (IGH), and 17p13 (TP53)].  ^d^Hyperdiploidy was defined as a gain of any two of the chromosomal regions 5p15, 9q22, and 15q22.^3^  ^e^High-risk chromosomal abnormalities (CA) were defined as del(17p), t(4;14), and t(14;16).^2^ Since t(4;14), and t(14;16) were not present in this subgroup, high-risk CA correspond to del(17p). ^f^Double-hit and triple-hit myeloma were defined as the co-occurrence of two or three adverse lesions, respectively.^4,5^ The translocations t(4;14), t(14;16),gain(1q) and del(17p) were counted as adverse; t(14;20) was not tested.  ^g^High-risk feature defined by Walker *et al.*^6^  ^h^Clinical feature at time of first sampling.  ^i^This category includes IgD, IgM, and non-secretory MM. ^j^This category includes alkylating agents and monoclonal antibodies. ^k^Only cases with a TTNT/OS >= 6 months (ASCT) or >= 12 months (maintenance) were considered. Hazard ratios (HR) and P values were derived using univariate Cox regression analysis. | | | | |

| **Table S8. Association between tetraploidy and cytogenetic, immunophenotypic and clinical features.** | | | | | | | | |
| --- | --- | --- | --- | --- | --- | --- | --- | --- |
| Variable | All cases | | Tetraploidy^a^ | | Early tetraploidy^a^ | | Late tetraploidy^a^ | |
| del(1p36) | 11/432 | 3% | 2/19 | 11% | 1/13 | 8% | 1/6 | 17% |
| del(1p32) | 32/362 | 9% | 4/11 | 36% | 3/10 | 30% | 1/1 | 100% |
| gain(1p32) *(≥ 3 copies)* | 20/362 | 6% | **6/11** | **55% **** | **6/10** | **60% **** | 0/1 | 0% |
| gain(1p36) *(≥ 3 copies)* | 22/432 | 5% | **12/19** | **63% **** | **12/13** | **92% **** | 0/6 | 0% |
| gain(1q) *(≥ 3 copies)* | 336/794 | 42% | **28/30** | **93% **** | **23/23** | **100% **** | 5/7 | 71% |
| del(11q) | 14/794 | 2% | 2/30 | 7% | 2/23 | 9% | 0/7 | 0% |
| gain(11q) *(≥ 3 copies)* | 375/794 | 47% | **25/30** | **83% **** | **21/23** | **91% **** | 4/7 | 57% |
| del(13q) | 356/794 | 45% | 13/30 | 43% | 7/23 | 30% | 6/7 | 86% |
| gain(13q) *(≥ 3 copies)* | 15/794 | 2% | **7/30** | **23% **** | **7/23** | **30% **** | 0/7 | 0% |
| del(14q) | 42/794 | 5% | 1/30 | 3% | 0/23 | 0% | 1/7 | 14% |
| Unspecified IGH alteration | 223/794 | 28% | 6/30 | 20% | 5/23 | 22% | 1/7 | 14% |
| del(17p) | 82/794 | 10% | 2/30 | 7% | 2/23 | 9% | 0/7 | 0% |
| gain(17p) *(≥ 3 copies)* | 80/794 | 10% | **20/30** | **67% **** | **20/23** | **87% **** | 0/7 | 0% |
| t(4;14) | 63/794 | 8% | 5/30 | 17% | 4/23 | 17% | 1/7 | 14% |
| t(11;14) | 146/794 | 18% | 7/30 | 23% | 4/23 | 17% | 3/7 | 43% |
| t(14;16) | 19/794 | 2% | **7/30** | **23% **** | **6/23** | **26% **** | 1/7 | 14% |
| Primary IGH translocations (i.e., t(4;14), t(11;14), or t(14;16)) | 228/794 | 29% | **19/30** | **63% *** | **14/23** | **61% *** | 5/7 | 71% |
| gain(1p32) *(≥ 4 copies)* | 7/362 | 2% | **4/11** | **36% **** | **4/10** | **40% **** | 0/1 | 0% |
| gain(1p36) *(≥ 4 copies)* | 9/432 | 2% | **8/19** | **42% **** | **8/13** | **62% **** | 0/6 | 0% |
| gain(1q) *(≥ 4 copies)* | 119/794 | 15% | **25/30** | **83% **** | **23/23** | **100% **** | 2/7 | 29% |
| gain(1q) *(≥ 5 copies)* | 30/794 | 4% | **9/30** | **30% **** | **7/23** | **30% **** | 2/7 | 29% |
| gain(11q) *(≥ 4 copies)* | 89/794 | 11% | **21/30** | **70% **** | **21/23** | **91% **** | 0/7 | 0% |
| gain(17p) *(≥ 4 copies)* | 21/794 | 3% | **20/30** | **67% **** | **20/23** | **87% **** | 0/7 | 0% |
| Hyperdiploidy^b^ | 65/119 | 55% | 6/6 | 100% | 6/6 | 100% | 0/0 | NaN% |
| High-risk CA^c^ | 158/794 | 20% | **13/30** | **43% *** | **11/23** | **48% *** | 2/7 | 29% |
| Double-hit^d^ | 87/794 | 11% | **13/30** | **43% **** | **11/23** | **48% **** | 2/7 | 29% |
| Triple-hit^d^ | 4/794 | 1% | 1/30 | 3% | 1/23 | 4% | 0/7 | 0% |
| gain(1q) (≥ 4 copies) plus ISS III^e^ | 20/236 | 8% | **6/16** | **38% *** | **6/10** | **60% **** | 0/6 | 0% |
| Female gender | 373/794 | 47% | 20/30 | 67% | 17/23 | 74% | 3/7 | 43% |
| Age (≥ 65 years)^f^ | 536/794 | 68% | 18/30 | 60% | 15/23 | 65% | 3/7 | 43% |
| BM plasma cell (≥ 60%)^f^ | 175/754 | 23% | **16/28** | **57% **** | **14/22** | **64% **** | 2/6 | 33% |
| Samples from pretreated patients | 135/794 | 17% | 4/30 | 13% | 3/23 | 13% | 1/7 | 14% |
| IgG | 203/365 | 56% | 10/20 | 50% | 6/13 | 46% | 4/7 | 57% |
| IgA | 81/365 | 22% | 5/20 | 25% | 4/13 | 31% | 1/7 | 14% |
| Light chain only | 76/365 | 21% | 4/20 | 20% | 2/13 | 15% | 2/7 | 29% |
| Other^g^ | 4/365 | 1% | 1/20 | 5% | 1/13 | 8% | 0/7 | 0% |
| Kappa | 239/370 | 65% | 13/22 | 59% | 9/15 | 60% | 4/7 | 57% |
| Lambda | 127/370 | 34% | 8/22 | 36% | 5/15 | 33% | 3/7 | 43% |
| LDH (increased) | 61/264 | 23% | 7/16 | 44% | 6/11 | 55% | 1/5 | 20% |
| Creatinine (≥ 2 mg/dL) | 46/273 | 17% | 5/17 | 29% | 3/11 | 27% | 2/6 | 33% |
| B2M (≥ 5.5 mg/L) | 74/247 | 30% | 7/14 | 50% | 5/9 | 56% | 2/5 | 40% |
| Hemoglobin (< 10 g/dL) | 85/247 | 34% | 8/16 | 50% | 6/11 | 55% | 2/5 | 40% |
| Platelets (< 150 G/L) | 57/249 | 23% | 4/15 | 27% | 3/10 | 30% | 1/5 | 20% |
| Calcium (≥ 2.75 mmol/L) | 17/260 | 7% | 2/16 | 12% | 1/11 | 9% | 1/5 | 20% |
| Involved/uninvolved SFLC ratio (≥ 100) | 91/222 | 41% | 8/14 | 57% | 5/8 | 62% | 3/6 | 50% |
| ISS I | 66/236 | 28% | 2/16 | 12% | 1/10 | 10% | 1/6 | 17% |
| ISS II | 96/236 | 41% | 6/16 | 38% | 3/10 | 30% | 3/6 | 50% |
| ISS III | 74/236 | 31% | 8/16 | 50% | 6/10 | 60% | 2/6 | 33% |
| R-ISS I | 42/219 | 19% | 1/14 | 7% | 1/9 | 11% | 0/5 | 0% |
| R-ISS II | 144/219 | 66% | 9/14 | 64% | 4/9 | 44% | 5/5 | 100% |
| R-ISS III | 33/219 | 15% | 4/14 | 29% | 4/9 | 44% | 0/5 | 0% |
| PI-based induction | 123/311 | 40% | 8/18 | 44% | 6/12 | 50% | 2/6 | 33% |
| IMiD-based induction | 14/311 | 5% | 2/18 | 11% | 2/12 | 17% | 0/6 | 0% |
| PI- and IMiD-based induction | 165/311 | 53% | 8/18 | 44% | 4/12 | 33% | 4/6 | 67% |
| Other^h^ | 9/311 | 3% | 0/18 | 0% | 0/12 | 0% | 0/6 | 0% |
| Maintenance/Consolidation^i^ | 82/181 | 45% | 8/9 | 89% | 5/6 | 83% | 3/3 | 100% |
| Front-line ASCT^i^ | 119/233 | 51% | 3/11 | 27% | 1/6 | 17% | 2/5 | 40% |
| Lines of therapy (≥3) | 99/287 | 34% | 8/17 | 47% | 4/11 | 36% | 4/6 | 67% |
| EMM^j^ | 52/328 | 16% | 7/20 | 35% | 4/14 | 29% | 3/6 | 50% |
| PCL^k^ | 24/328 | 7% | 3/20 | 15% | 3/14 | 21% | 0/6 | 0% |
| AL | 14/323 | 4% | 1/19 | 5% | 0/13 | 0% | 1/6 | 17% |
| CD38 | 317/318 | 100% | 17/17 | 100% | 12/12 | 100% | 5/5 | 100% |
| CD138 | 269/278 | 97% | 14/15 | 93% | 9/10 | 90% | 5/5 | 100% |
| CD45 | 36/235 | 15% | 4/14 | 29% | 4/10 | 40% | 0/4 | 0% |
| CD19 | 6/312 | 2% | 0/16 | 0% | 0/11 | 0% | 0/5 | 0% |
| CD117 | 108/255 | 42% | 7/14 | 50% | 6/10 | 60% | 1/4 | 25% |
| CD56 | 190/280 | 68% | 8/16 | 50% | 5/11 | 45% | 3/5 | 60% |
| CD27 | 88/149 | 59% | 5/9 | 56% | 4/6 | 67% | 1/3 | 33% |
| CD28 | 41/135 | 30% | 3/6 | 50% | 1/4 | 25% | 2/2 | 100% |
| CD81 | 47/147 | 32% | 4/10 | 40% | 2/7 | 29% | 2/3 | 67% |
| Co-expression CD56/CD117 | 72/303 | 24% | 3/17 | 18% | 3/12 | 25% | 0/5 | 0% |

*Note:* Clinical features at diagnosis, unless otherwise indicated.

Abbreviations: AL, amyloidosis; ASCT, autologous stem cell transplantation; BM, bone marrow; B2M, beta-2 microglobulin; IMiD, immunomodulatory drugs; ISS, International Staging System^1^; LDH, lactate dehydrogenase; PI, proteasome inhibitors; R-ISS, revised International Staging System^2^; SFLC, serum free light chain.
^a^Tetraploidy was defined as three or more amplified regions (four or more copies) with the standard FISH panel [1p36 (D1S2795,D1S253) or 1p32 (CDKN2C), 1q21 (CKS1B), 11q22 (ATM), 13q14 (DLEU1), 14q32 (IGH), and 17p13 (TP53)]. If tetraploidy was early, the tetraploid clone was detectable already in the first analyzed sample and if tetraploidy was late, the tetraploid clone was detectable only in a subsequent sample.

^b^Hyperdiploidy was defined as a gain of any two of the chromosomal regions 5p15, 9q22, and 15q22.^3^
^c^High-risk chromosomal abnormalities (CA) were defined as del(17p), t(4;14), and t(14;16).^2^
^d^Double-hit and triple-hit myeloma were defined as the co-occurrence of two or three adverse lesions, respectively.^4,5^ The translocations t(4;14), t(14;16),gain(1q) and del(17p) were counted as adverse; t(14;20) was not tested.

^e^High-risk feature defined by Walker *et al.*^6^

^f^Clinical feature at time of first sampling.
^g^This category includes IgD, IgM, and non-secretory MM.
^h^This category includes alkylating agents and monoclonal antibodies.

^i^Only cases with a TTNT/OS >= 6 months (ASCT) or >= 12 months (maintenance) were considered.

^j^Extramedullary myeloma (EMM) was present at diagnosis or developed during disease course and was defined as plasma cell infiltration of the soft tissue (extramedullary extraosseous and/or extramedullary bone-related).
^k^Plasma cell leukemia (PCL) was present at diagnosis or developed during disease course.

*P < 0.05; **P < 0.001, Fisher’s exact test. For multiple testing *P* values were adjusted with the Benjamini-Hochberg method.

| **Table S9. Association between myeloma having an extramedullary manifestation and cytogenetic and clinical features.** | | | | | | | | | | | | | | |
| --- | --- | --- | --- | --- | --- | --- | --- | --- | --- | --- | --- | --- | --- | --- |
| Variable | All cases | | EMM^a^ | | pEMM^a^ | | sEMM^a^ | | PCL^b^ | | pPCL^b^ | | sPCL^b^ | |
| del(1p36) | 3/210 | 1% | 0/39 | 0% | 0/15 | 0% | 0/24 | 0% | 0/18 | 0% | 0/9 | 0% | 0/9 | 0% |
| del(1p32) | 10/118 | 8% | 2/13 | 15% | 2/11 | 18% | 0/2 | 0% | 1/6 | 17% | 1/4 | 25% | 0/2 | 0% |
| gain(1p32) *(≥ 3 copies)* | 8/118 | 7% | 1/13 | 8% | 0/11 | 0% | 1/2 | 50% | 2/6 | 33% | 1/4 | 25% | 1/2 | 50% |
| gain(1p36) *(≥ 3 copies)* | 15/210 | 7% | 3/39 | 8% | 1/15 | 7% | 2/24 | 8% | 3/18 | 17% | 1/9 | 11% | 2/9 | 22% |
| gain(1q) *(≥ 3 copies)* | 160/328 | 49% | 31/52 | 60% | 12/26 | 46% | 19/26 | 73% | 13/24 | 54% | 6/13 | 46% | 7/11 | 64% |
| del(11q) | 6/328 | 2% | 1/52 | 2% | 1/26 | 4% | 0/26 | 0% | 0/24 | 0% | 0/13 | 0% | 0/11 | 0% |
| gain(11q) *(≥ 3 copies)* | 159/328 | 48% | 26/52 | 50% | 12/26 | 46% | 14/26 | 54% | 11/24 | 46% | 7/13 | 54% | 4/11 | 36% |
| del(13q) | 164/328 | 50% | 28/52 | 54% | 13/26 | 50% | 15/26 | 58% | 16/24 | 67% | 9/13 | 69% | 7/11 | 64% |
| gain(13q) *(≥ 3 copies)* | 9/328 | 3% | 1/52 | 2% | 0/26 | 0% | 1/26 | 4% | 1/24 | 4% | 1/13 | 8% | 0/11 | 0% |
| del(14q) | 15/328 | 5% | 1/52 | 2% | 0/26 | 0% | 1/26 | 4% | 1/24 | 4% | 1/13 | 8% | 0/11 | 0% |
| Unspecified IGH alteration | 90/328 | 27% | 11/52 | 21% | 6/26 | 23% | 5/26 | 19% | 5/24 | 21% | 4/13 | 31% | 1/11 | 9% |
| del(17p) | 29/328 | 9% | 5/52 | 10% | 2/26 | 8% | 3/26 | 12% | 6/24 | 25% | **5/13** | **38% *** | 1/11 | 9% |
| gain(17p) *(≥ 3 copies)* | 38/328 | 12% | 7/52 | 13% | 3/26 | 12% | 4/26 | 15% | 4/24 | 17% | 2/13 | 15% | 2/11 | 18% |
| t(4;14) | 33/328 | 10% | 7/52 | 13% | 2/26 | 8% | 5/26 | 19% | 5/24 | 21% | 1/13 | 8% | 4/11 | 36% |
| t(11;14) | 63/328 | 19% | 8/52 | 15% | 6/26 | 23% | 2/26 | 8% | 7/24 | 29% | 3/13 | 23% | 4/11 | 36% |
| t(14;16) | 9/328 | 3% | 2/52 | 4% | 0/26 | 0% | 2/26 | 8% | **4/24** | **17% *** | 2/13 | 15% | 2/11 | 18% |
| IGH tr. (t(4;14), t(11;14), or t(14;16)) | 105/328 | 32% | 17/52 | 33% | 8/26 | 31% | 9/26 | 35% | **16/24** | **67% *** | 6/13 | 46% | **10/11** | **91% *** |
| gain(1p32) *(≥ 4 copies)* | 3/118 | 3% | 0/13 | 0% | 0/11 | 0% | 0/2 | 0% | 0/6 | 0% | 0/4 | 0% | 0/2 | 0% |
| gain(1p36) *(≥ 4 copies)* | 8/210 | 4% | 2/39 | 5% | 1/15 | 7% | 1/24 | 4% | 2/18 | 11% | 1/9 | 11% | 1/9 | 11% |
| gain(1q) *(≥ 4 copies)* | 56/328 | 17% | 13/52 | 25% | 5/26 | 19% | 8/26 | 31% | 5/24 | 21% | 3/13 | 23% | 2/11 | 18% |
| gain(1q) *(≥ 5 copies)* | 18/328 | 5% | 4/52 | 8% | 3/26 | 12% | 1/26 | 4% | 2/24 | 8% | 2/13 | 15% | 0/11 | 0% |
| gain(11q) *(≥ 4 copies)* | 38/328 | 12% | 9/52 | 17% | 2/26 | 8% | 7/26 | 27% | 5/24 | 21% | 3/13 | 23% | 2/11 | 18% |
| gain(17p) *(≥ 4 copies)* | 12/328 | 4% | 3/52 | 6% | 1/26 | 4% | 2/26 | 8% | 4/24 | 17% | 2/13 | 15% | 2/11 | 18% |
| Tetraploidy^c^ | 20/328 | 6% | 7/52 | 13% | 1/26 | 4% | **6/26** | **23% *** | 3/24 | 12% | 1/13 | 8% | 2/11 | 18% |
| Early tetraploidy^c^ | 14/328 | 4% | 4/52 | 8% | 1/26 | 4% | 3/26 | 12% | 3/24 | 12% | 1/13 | 8% | 2/11 | 18% |
| Late tetraploidy^c^ | 6/328 | 2% | 3/52 | 6% | 0/26 | 0% | 3/26 | 12% | 0/24 | 0% | 0/13 | 0% | 0/11 | 0% |
| Hyperdiploidy^d^ | 18/36 | 50% | 2/5 | 40% | 1/4 | 25% | 1/1 | 100% | 1/4 | 25% | 0/2 | 0% | 1/2 | 50% |
| Double-hit^e^ | 40/328 | 12% | 6/52 | 12% | 1/26 | 4% | 5/26 | 19% | **10/24** | **42% *** | 4/13 | 31% | **6/11** | **55% *** |
| Triple-hit^e^ | 2/328 | 1% | 2/52 | 4% | 1/26 | 4% | 1/26 | 4% | 0/24 | 0% | 0/13 | 0% | 0/11 | 0% |
| High-risk CA^f^ | 69/328 | 21% | 12/52 | 23% | 3/26 | 12% | 9/26 | 35% | **15/24** | **62% **** | **8/13** | **62% *** | **7/11** | **64% *** |
| gain(1q) (≥ 4 copies) plus ISS III^g^ | 18/211 | 9% | 2/39 | 5% | 1/20 | 5% | 1/19 | 5% | 1/13 | 8% | 1/6 | 17% | 0/7 | 0% |
| Female gender | 144/328 | 44% | 22/52 | 42% | 12/26 | 46% | 10/26 | 38% | 14/24 | 58% | 5/13 | 38% | 9/11 | 82% |
| Age (≥ 65 years)^h^ | 185/328 | 56% | 26/52 | 50% | 15/26 | 58% | 11/26 | 42% | 15/24 | 62% | 10/13 | 77% | 5/11 | 45% |
| BM plasma cell (≥ 60%)^h^ | 80/309 | 26% | 13/46 | 28% | 4/22 | 18% | 9/24 | 38% | 11/23 | 48% | 8/13 | 62% | 3/10 | 30% |
| Samples from pretreated patients | 57/328 | 17% | 13/52 | 25% | 3/26 | 12% | 10/26 | 38% | 1/24 | 4% | 0/13 | 0% | 1/11 | 9% |
| IgG | 171/308 | 56% | 30/49 | 61% | 16/23 | 70% | 14/26 | 54% | 11/20 | 55% | 6/11 | 55% | 5/9 | 56% |
| IgA | 65/308 | 21% | 9/49 | 18% | 3/23 | 13% | 6/26 | 23% | 2/20 | 10% | 1/11 | 9% | 1/9 | 11% |
| Light chain only | 68/308 | 22% | 9/49 | 18% | 3/23 | 13% | 6/26 | 23% | 6/20 | 30% | 4/11 | 36% | 2/9 | 22% |
| Other^i^ | 4/308 | 1% | 1/49 | 2% | 1/23 | 4% | 0/26 | 0% | 1/20 | 5% | 0/11 | 0% | 1/9 | 11% |
| Kappa | 196/309 | 63% | 32/51 | 63% | 15/25 | 60% | 17/26 | 65% | 12/20 | 60% | 7/11 | 64% | 5/9 | 56% |
| Lambda | 110/309 | 36% | 19/51 | 37% | 9/25 | 36% | 10/26 | 38% | 7/20 | 35% | 4/11 | 36% | 3/9 | 33% |
| LDH (increased) | 56/238 | 24% | 12/36 | 33% | 5/16 | 31% | 7/20 | 35% | 2/16 | 12% | 1/8 | 12% | 1/8 | 12% |
| Creatinine (≥ 2 mg/dL) | 42/247 | 17% | 5/38 | 13% | 2/18 | 11% | 3/20 | 15% | 3/16 | 19% | 3/8 | 38% | 0/8 | 0% |
| B2M (≥ 5.5 mg/L) | 64/221 | 29% | 10/33 | 30% | 6/18 | 33% | 4/15 | 27% | 7/12 | 58% | 3/5 | 60% | 4/7 | 57% |
| Hemoglobin (< 10 g/dL) | 76/222 | 34% | 14/33 | 42% | 5/15 | 33% | 9/18 | 50% | 10/18 | 56% | **8/10** | **80% *** | 2/8 | 25% |
| Platelets (< 150 G/L) | 50/223 | 22% | 4/32 | 12% | 2/14 | 14% | 2/18 | 11% | **14/17** | **82% **** | **8/9** | **89% **** | **6/8** | **75% *** |
| Calcium (≥ 2.75 mmol/L) | 16/235 | 7% | 2/34 | 6% | 1/16 | 6% | 1/18 | 6% | 1/16 | 6% | 1/8 | 12% | 0/8 | 0% |
| Involved/uninvolved SFLC ratio (≥ 100) | 78/197 | 40% | 12/30 | 40% | 5/13 | 38% | 7/17 | 41% | 8/15 | 53% | 4/9 | 44% | 4/6 | 67% |
| ISS I | 59/211 | 28% | 10/39 | 26% | 6/20 | 30% | 4/19 | 21% | 1/13 | 8% | 1/6 | 17% | 0/7 | 0% |
| ISS II | 88/211 | 42% | 17/39 | 44% | 7/20 | 35% | 10/19 | 53% | 5/13 | 38% | 1/6 | 17% | 4/7 | 57% |
| ISS III | 64/211 | 30% | 12/39 | 31% | 7/20 | 35% | 5/19 | 26% | 7/13 | 54% | 4/6 | 67% | 3/7 | 43% |
| R-ISS I | 38/196 | 19% | 3/27 | 11% | 3/13 | 23% | 0/14 | 0% | 0/12 | 0% | 0/5 | 0% | 0/7 | 0% |
| R-ISS II | 129/196 | 66% | 19/27 | 70% | 6/13 | 46% | 13/14 | 93% | 9/12 | 75% | 4/5 | 80% | 5/7 | 71% |
| R-ISS III | 29/196 | 15% | 5/27 | 19% | 4/13 | 31% | 1/14 | 7% | 3/12 | 25% | 1/5 | 20% | 2/7 | 29% |
| PI-based induction | 119/305 | 39% | 20/50 | 40% | 12/25 | 48% | 8/25 | 32% | 5/19 | 26% | 2/10 | 20% | 3/9 | 33% |
| IMiD-based induction | 14/305 | 5% | 5/50 | 10% | 1/25 | 4% | 4/25 | 16% | 0/19 | 0% | 0/10 | 0% | 0/9 | 0% |
| PI- and IMiD-based induction | 163/305 | 53% | 25/50 | 50% | 12/25 | 48% | 13/25 | 52% | 14/19 | 74% | 8/10 | 80% | 6/9 | 67% |
| Other^j^ | 9/305 | 3% | 0/50 | 0% | 0/25 | 0% | 0/25 | 0% | 0/19 | 0% | 0/10 | 0% | 0/9 | 0% |
| Maintenance^k^ | 81/179 | 45% | 10/26 | 38% | 5/12 | 42% | 5/14 | 36% | 2/5 | 40% | 1/3 | 33% | 1/2 | 50% |
| Front-line ASCT^k^ | 119/230 | 52% | 23/37 | 62% | 11/17 | 65% | 12/20 | 60% | 5/12 | 42% | 2/5 | 40% | 3/7 | 43% |
| Lines of therapy (≥3) | 98/283 | 35% | **27/47** | **57% *** | 8/22 | 36% | **19/25** | **76% **** | 6/18 | 33% | 0/10 | 0% | 6/8 | 75% |
| EMM^a^ | 52/328 | 16% | NA | NA | **26/26** | **100% **** | **26/26** | **100% **** | 2/24 | 8% | 1/13 | 8% | 1/11 | 9% |
| pEMM^a^ | 26/328 | 8% | **26/52** | **50% **** | NA | NA | 0/26 | 0% | 1/24 | 4% | 1/13 | 8% | 0/11 | 0% |
| sEMM^a^ | 26/328 | 8% | **26/52** | **50% **** | 0/26 | 0% | NA | NA | 1/24 | 4% | 0/13 | 0% | 1/11 | 9% |
| PCL^b^ | 24/328 | 7% | 2/52 | 4% | 1/26 | 4% | 1/26 | 4% | NA | NA | **13/13** | **100% **** | **11/11** | **100% **** |
| pPCL^b^ | 13/328 | 4% | 1/52 | 2% | 1/26 | 4% | 0/26 | 0% | **13/24** | **54% **** | NA | NA | 0/11 | 0% |
| sPCL^b^ | 11/328 | 3% | 1/52 | 2% | 0/26 | 0% | 1/26 | 4% | **11/24** | **46% **** | 0/13 | 0% | NA | NA |
| AL | 13/321 | 4% | 2/50 | 4% | 0/24 | 0% | 2/26 | 8% | 0/20 | 0% | 0/11 | 0% | 0/9 | 0% |
| CD38 | 233/234 | 100% | 30/30 | 100% | 13/13 | 100% | 17/17 | 100% | 19/19 | 100% | 10/10 | 100% | 9/9 | 100% |
| CD138 | 203/207 | 98% | 25/26 | 96% | 13/13 | 100% | 12/13 | 92% | 18/18 | 100% | 9/9 | 100% | 9/9 | 100% |
| CD45 | 30/181 | 17% | 6/23 | 26% | 3/10 | 30% | 3/13 | 23% | 3/18 | 17% | 1/10 | 10% | 2/8 | 25% |
| CD19 | 3/231 | 1% | 1/31 | 3% | 0/14 | 0% | 1/17 | 6% | 0/19 | 0% | 0/11 | 0% | 0/8 | 0% |
| CD117 | 75/187 | 40% | 3/21 | 14% | 1/9 | 11% | 2/12 | 17% | 2/16 | 12% | 1/8 | 12% | 1/8 | 12% |
| CD56 | 142/205 | 69% | 19/27 | 70% | 9/11 | 82% | 10/16 | 62% | 6/14 | 43% | 3/6 | 50% | 3/8 | 38% |
| CD27 | 59/104 | 57% | 4/12 | 33% | 2/6 | 33% | 2/6 | 33% | 2/7 | 29% | 2/5 | 40% | 0/2 | 0% |
| CD28 | 35/102 | 34% | 3/8 | 38% | 0/4 | 0% | 3/4 | 75% | 2/7 | 29% | 1/5 | 20% | 1/2 | 50% |
| CD81 | 29/106 | 27% | 4/10 | 40% | 1/6 | 17% | 3/4 | 75% | 2/5 | 40% | 2/4 | 50% | 0/1 | 0% |
| Co-expression CD56/CD117 | 50/225 | 22% | 2/29 | 7% | 1/12 | 8% | 1/17 | 6% | 0/19 | 0% | 0/10 | 0% | 0/9 | 0% |

*Note:* Clinical features at diagnosis, unless otherwise indicated.

Abbreviations: AL, amyloidosis; ASCT, autologous stem cell transplantation; BM, bone marrow; B2M, beta-2 microglobulin; IMiD, immunomodulatory drugs; ISS, International Staging System^1^; LDH, lactate dehydrogenase; PI, proteasome inhibitors; R-ISS, revised International Staging System^2^; SFLC, serum free light chain.

^a^Extramedullary myeloma (EMM) was present at diagnosis (primary EMM, pEMM) or developed during disease course (secondary EMM, sEMM) and was defined as plasma cell infiltration of the soft tissue (extramedullary extraosseous and/or extramedullary bone-related).
^b^Plasma cell leukemia (PCL) was present at diagnosis (primary PCL, pPCL) or developed during disease course (secondary PCL, sPCL).
^c^Tetraploidy was defined as three or more amplified (four or more copies) regions with the standard FISH panel [1p36 (D1S2795,D1S253) or 1p32 (CDKN2C), 1q21 (CKS1B), 11q22 (ATM), 13q14 (DLEU1), 14q32 (IGH), and 17p13 (TP53)]. If tetraploidy was early, the tetraploid clone was detectable already in the first analyzed sample and if tetraploidy was late, the tetraploid clone was detectable only in a subsequent sample.

^d^Hyperdiploidy was defined as a gain of any two of the chromosomal regions 5p15, 9q22, and 15q22.^3^

^e^Double-hit and triple-hit myeloma were defined as the co-occurrence of two or three adverse lesions, respectively.^4,5^ The translocations t(4;14), t(14;16),gain(1q) and del(17p) were counted as adverse; t(14;20) was not tested.

^f^High-risk chromosomal abnormalities (CA) were defined as del(17p), t(4;14), and t(14;16).^2^
^g^High-risk feature defined by Walker et al*.*^6^

^h^Clinical feature at time of first sampling.
^i^This category includes IgD, IgM, and non-secretory MM.
^j^This category includes alkylating agents and monoclonal antibodies.
^k^Only cases with a TTNT/OS >= 6 months (ASCT) or >= 12 months (maintenance) were considered.

*P < 0.05; **P < 0.001, Fisher’s exact test. For multiple testing *P* values were adjusted with the Benjamini-Hochberg method.

**References**

1. Greipp PR, Miguel JS, Dune BGM, et al. International staging system for multiple myeloma. *J Clin Oncol*. 2005;23(15):3412-3420. doi:10.1200/JCO.2005.04.242

2. Palumbo A, Avet-Loiseau H, Oliva S, et al. Revised international staging system for multiple myeloma: A report from international myeloma working group. *J Clin Oncol*. 2015;33(26):2863-2869. doi:10.1200/JCO.2015.61.2267

3. Wuilleme S, Robillard N, Lodé L, et al. Ploidy, as detected by fluorescence in situ hybridization, defines different subgroups in multiple myeloma. *Leukemia*. 2005;19(2):275-278. doi:10.1038/sj.leu.2403586

4. Shah V, Sherborne AL, Walker BA, et al. Prediction of outcome in newly diagnosed myeloma: a meta-analysis of the molecular profiles of 1905 trial patients. *Leukemia*. 2018;32(1):102-110. doi:10.1038/leu.2017.179

5. Rajkumar SV. Multiple myeloma: 2020 update on diagnosis, risk-stratification and management. *Am J Hematol*. 2020;95(5):548-567. doi:10.1002/ajh.25791

6. Walker BA, Mavrommatis K, Wardell CP, et al. A high-risk, Double-Hit, group of newly diagnosed myeloma identified by genomic analysis. *Leukemia*. 2019;33(1):159-170. doi:10.1038/s41375-018-0196-8
